# Comprehensive Evaluation of Organotypic and Microphysiological Liver Models for Prediction of Drug-Induced Liver Injury

**DOI:** 10.3389/fphar.2019.01093

**Published:** 2019-09-24

**Authors:** Yitian Zhou, Joanne X. Shen, Volker M. Lauschke

**Affiliations:** Department of Physiology and Pharmacology, Karolinska Institutet, Stockholm, Sweden

**Keywords:** hepatotoxicity, 3D cell culture, spheroids, liver-on-a-chip, bioreactors

## Abstract

Drug-induced liver injury (DILI) is a major concern for the pharmaceutical industry and constitutes one of the most important reasons for the termination of promising drug development projects. Reliable prediction of DILI liability in preclinical stages is difficult, as current experimental model systems do not accurately reflect the molecular phenotype and functionality of the human liver. As a result, multiple drugs that passed preclinical safety evaluations failed due to liver toxicity in clinical trials or postmarketing stages in recent years. To improve the selection of molecules that are taken forward into the clinics, the development of more predictive *in vitro* systems that enable high-throughput screening of hepatotoxic liabilities and allow for investigative studies into DILI mechanisms has gained growing interest. Specifically, it became increasingly clear that the choice of cell types and culture method both constitute important parameters that affect the predictive power of test systems. In this review, we present current 3D culture paradigms for hepatotoxicity tests and critically evaluate their utility and performance for DILI prediction. In addition, we highlight possibilities of these emerging platforms for mechanistic evaluations of selected drug candidates and present current research directions towards the further improvement of preclinical liver safety tests. We conclude that organotypic and microphysiological liver systems have provided an important step towards more reliable DILI prediction. Furthermore, we expect that the increasing availability of comprehensive benchmarking studies will facilitate model dissemination that might eventually result in their regulatory acceptance.

## Introduction

Drug-induced liver injury (DILI) constitutes a rare life-threatening adverse drug reaction (ADR). While it only occurs in 4–19 per 100,000 individuals, it is nevertheless the most common cause for acute liver failure in the US and Europe (Sgro et al., 2002; Bjornsson et al., 2013). Of the affected patients, 9.4% die or require liver transplantation and 18.9% show persistent liver damage 6 months after DILI diagnosis (Fontana et al., 2014). Acetaminophen overdose is the most common cause of acute liver failure, whereas around 13% of DILI cases in the clinics are idiosyncratic (Ostapowicz et al., 2002). In addition to its clinical importance, DILI constitutes a major problem for the pharmaceutical industry and regulatory authorities. In the last 30 years, 14 drugs have been withdrawn from the US and European markets due to hepatotoxicity in post-marketing stages (**Table 1**). Withdrawal of medications represents a major financial burden for the pharmaceutical industry. An impressive example is provided by the later withdrawn antidiabetic drug troglitazone, which achieved sales of 750 million USD per year in 1998; however, when regulators in the UK announced that risks of troglitazone therapy outweighed benefits, share prices of the manufacturer Warner-Lambert dropped by 18.5%, corresponding to a loss of approximately 10 billion USD in company value (Gale, 2006). In addition to post-marketing withdrawals, DILI was responsible for the discontinuation of numerous drug development programs across all clinical stages, including the phase 3 terminations of aplaviroc (Nichols et al., 2008) and fasiglifam (Marcinak et al., 2018).

**Table 1:** Drugs and drug candidates that were withdrawn from US and major European markets due to hepatotoxicity since 1990.

Drug	Drug class	Chemical taxonomy	Fraction of patients with liver test abnormalities	Year	Reference
Dilevalol	Antihypertensive	Salicylamide	2%	1990	(Chrisp and Goa, 1990)
Pirprofen	NSAID	Propionic acid	Case reports	1990	–
Bendazac	Ophthalmologic	Oxyacetic acid	Case reports	1993	–
Alpidem	Anxiolytic	Imidazopyridine	Case reports	1994	–
Tolrestat	Management of diabetic complications	Naphthalen	Case reports	1996	(Foppiano and Lombardo, 1997)
Bromfenac*	NSAID	Benzophenone	6%	1998	(Goldkind and Laine, 2006)
Tolcapone	Dopaminergic	Benzophenone	1–4%	1998	(Olanow and Panel, 2000)
Troglitazone	Antidiabetic	Thiazolidinedione	2%	2000	(Graham et al., 2001)
Nefazodone	Antidepressant	Phenylpiperazine	29 cases per 100,000 patient years	2003	(Spigset et al., 2003)
Pemoline	Psychostimulant	4-oxazolidinone	1–3%	2005	(Safer et al., 2001)
Ximelagatran	Antihypertensive	Dipeptide	0.8–4%	2006	(Francis et al., 2003; Petersen et al., 2003)
Lumiracoxib	NSAID	Aminotoluene	3%	2007	(Schnitzer et al., 2004)
Sitaxentan	Antihypertensive	Benzodioxole	6%	2010	(Benza et al., 2008)
Daclizumab	Interleukin inhibitor	Monoclonal antibody	6%	2018	(Kappos et al., 2015)

*Withdrawal pertains to the oral formulation. Bromfenac is still marketed as an ophthalmic solution.

NSAID, non-steroidal anti-inflammatory drug. Note that drugs that were only withdrawn in individual European countries are not listed here.

In light of this preamble, it is clear that the assessment of potential safety liabilities is an essential element of preclinical drug development in order to reduce the risk of expensive late project failures. Importantly, projects with preclinical safety signals are often closed in the clinic due to safety issues, emphasizing the need to thoroughly evaluate and act upon preclinical safety profiles (Cook et al., 2014). The mandatory core battery of pharmacological safety tests is formalized in guidelines by the International Conference on Harmonization (ICH) of Technical Requirements for Registration of Pharmaceuticals for Human Use and is implemented in Europe (EC), Japan (PMDA), US (FDA), Canada (Health Canada), and Switzerland (Swissmedic). Notably, these directives only specify tests covering central nervous, cardiovascular, and respiratory toxicity as mandatory (https://www.ich.org/products/guidelines/safety/article/safety-guidelines.html), whereas evaluation of DILI liability is not explicitly required. In 2010, however, EMEA has released a white paper to assess the risk of DILI during preclinical stages (https://www.ema.europa.eu/en/documents/scientific-guideline/reflection-paper-non-clinical-evaluation-drug-induced-liver-injury-dili_en.pdf), and these developments can be interpreted as the intention of regulators to formalize specific guidance for DILI in the near future.

Before first-in-human trials, drug safety is evaluated in preclinical animal models. However, 38–51% of compounds with hepatotoxic liabilities are not detected in preclinical tests (Hughes, 2008), and the development of systems that more faithfully capture human liver toxicity is thus a major focus of both academic research and commercial development. Early attempts to test and benchmark methods for their ability to predict human toxicity were carried out in the 1990s in the frame of the Multicentre Evaluation of *In Vitro* Cytotoxicity (MEIC) project (Bondesson et al., 1989). In the course of this project, 29 laboratories tested the cytotoxicity of 50 reference chemicals in 61 cell models, including the hepatoma cell line HepG2. Importantly, the authors found that human cell lines outperformed animal cell culture systems for the prediction of human lethal peak concentrations (Ekwall et al., 1998), thus setting the stage for the adoption of human cell models for toxicity studies.

Fueled by improvements in hepatocyte isolation methods and cryopreservation protocols, as well as the increasing appreciation that hepatoma cell lines express only very low levels of drug metabolizing enzymes, the focus shifted to primary human hepatocytes (PHH) as the gold standard cell model for predictive toxicology. While freshly isolated PHH closely resemble hepatocytes *in situ*, their phenotype rapidly deteriorates in conventional 2D culture in a process called dedifferentiation. Earliest perturbations on transcript level are already apparent after 30 min and more than 4,000 transcripts are differentially expressed (FDR = 0.01) during the first 24 h of culture (Lauschke et al., 2016). Significantly affected pathways include tricarboxylic acid (TCA) cycle, oxidative phosphorylation, fatty acid metabolism, and urea synthesis (Lauschke et al., 2016). Furthermore, 2D cultured PHH lose expression of important drug metabolizing enzymes and drug transporters, which hampers their usefulness for studies of liver biology and pathobiology, as well as for drug metabolism and toxicity (Rowe et al., 2013).

To overcome these problems, PHH can be cultured in sandwich configuration in which 2D cultured hepatocytes are overlaid with an additional layer of Matrigel. In this culture paradigm PHH retain their morphology and polarity, resulting in physiologically relevant expression levels of transporters and the formation of functional bile canaliculi networks (Bi et al., 2006; Swift et al., 2010; De Bruyn et al., 2013; Oorts et al., 2016). While longevity of cells is prolonged in this culture configuration, proteomic analyses revealed clear indications of hepatocyte dedifferentiation and declining expression levels of multiple CYPs in sandwich cultures (Kimoto et al., 2012; Rowe et al., 2013; Bell et al., 2018). In acute toxicity studies, sandwich-cultured PHH achieved a sensitivity of 50–60% with a specificity of 100% (n = 200 DILI positive and n = 144 DILI negative compounds) (Xu et al., 2008). Underlying reasons for the high number of false negatives might be the limited mass transfer of compounds through the extracellular matrix (ECM) overlay and the fact that cells in 2D sandwich culture cannot be cultivated long enough to mimic the delayed onset of many hepatotoxic compounds. Thus, while PHH sandwich cultures provide useful tools for pharmacological studies, particularly for the analysis of hepatobiliary transport and cholestatic toxicity, they are only of limited utility for the mechanism-agnostic screening of liver toxicity.

In an attempt to improve phenotypic maintenance of liver cells in culture, a vast number of 3D culture methods have been developed during recent years (Lauschke et al., 2016; Lin and Khetani, 2016; Underhill and Khetani, 2018; Lauschke et al., 2019). In the following sections we outline important considerations for the development of such organotypic liver models, and reflect on how 3D culture systems can promote the maintenance of differentiation characteristics of the parent organ. Next, we provide an updated comprehensive overview of current hepatic organotypic and microphysiological *in vitro* models for the prediction of DILI, including spheroid models, micropatterned co-cultures (MPCC), bioreactors, and liver-on-a-chip devices, and critically discuss their strengths and weaknesses. In the last part of the review, we highlight emerging applications beyond the evaluation of hepatotoxicity for which organotypic liver models promise to provide conceptual advancements over conventional 2D cultures.

## Features of Organotypic Liver Models

**Figure 1:**
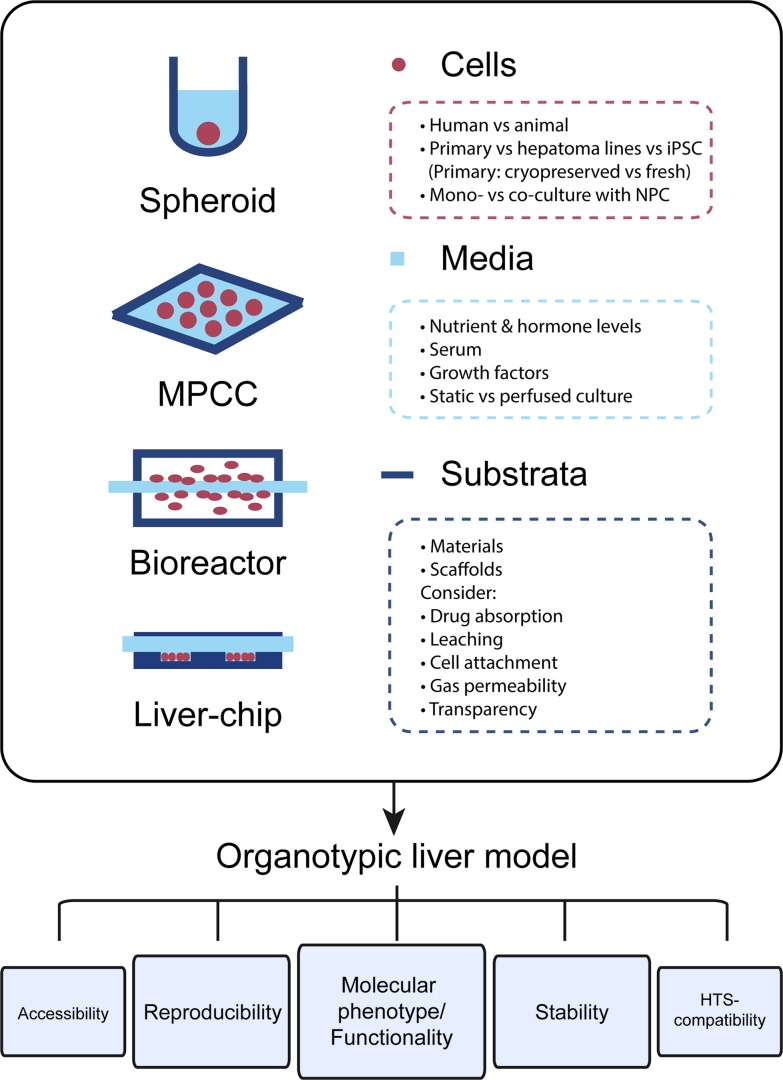
The choice of cells, media, and culture substrata markedly influences the phenotype and functional relevance of organotypic liver models. Important points that should be considered for model design are listed. Each newly established model should closely mimic the molecular phenotype and function of human liver tissue. Furthermore, cellular phenotypes should be stable over the course of multiple weeks in culture and assays should be reproducible, i.e., experiments using cells from the same donor should have low technical variability. Further ideal organotypic liver models should be sufficiently accessible, allowing time course measurements and the sampling of various end points, and should be compatible with high-throughput screening (HTS).

The most important feature of organotypic liver models is the preservation of robust hepatic functions and physiological expression levels of drug metabolizing enzymes and drug transporters in order to facilitate faithful hepatotoxicity predictions (**Figure 1**). For this reason, PHH cultures are more predictive than hepatoma cell lines, such as HepG2, which exhibit expression levels of drug metabolizing enzymes that are often orders of magnitude lower than in freshly isolated PHH (Donato et al., 2009). Importantly, PHH can be cryopreserved and maintain their metabolic activity and sensitivity to drugs upon thawing, which allows biologically meaningful replicate experiments using material from the same donor (McGinnity et al., 2004; Hallifax et al., 2008; Sison-Young et al., 2017). Furthermore, hepatic models should capture the human liver *in vivo* microenvironment, including the phenotypes of and interactions with relevant non-parenchymal cells (NPCs). Additionally, viability and molecular signatures of the cultured cells should be stable. As such, it is crucial that the rapid dedifferentiation observed within hours in 2D monolayer PHH cultures is prevented (Lauschke et al., 2016). This is particularly important for assessments of drug hepatotoxicity, as toxicity for the majority of compounds manifests with a delayed onset and, additionally, drug metabolizing enzymes are among the first to be lost during dedifferentiation.

In addition to the utilized cell models and culture paradigms, the composition of the culture medium has strong influence on both the phenotypes and stability of the cell model of choice. Specific consideration should be given to nutrient concentrations (sugars, amino acids, and vitamins) and hormone levels, most importantly insulin. Furthermore, while the use of serum might be supporting overall cell health, its use can complicate result interpretation due to incomplete information regarding its active constituents. Similar arguments apply to the use of media with proprietary non-disclosed formulations.

Lastly, the influence of substratum materials and, where applicable, scaffolds on the bioavailability of tested compounds should also be noted. Particularly, it is crucial that these systems exhibit low absorption of small molecules. This issue of drug absorption limits the predictive power of many culture systems, particularly those relying on the elastomer polydimethylsiloxane (PDMS) (Toepke and Beebe, 2006; Li et al., 2009; Gomèz-Sjoberg et al., 2010; Wang et al., 2012). In addition, the material of choice should exhibit minimal leaching of uncrosslinked oligomers into the culture medium, which can be bioactive and modulate drug action (McDonald et al., 2008). Oxygen concentrations in the medium can influence the molecular phenotype of hepatic cells, and it is thus important that environmental oxygen concentrations, platform design, and gas permeability of the substratum material are harmonized to guarantee that cells are exposed to relevant oxygen levels. Another key parameter for liver chips is the stiffness and topography of the substratum material, which can affect molecular phenotypes of cultured hepatocytes *via* mechanosensing pathways (Natarajan et al., 2015; Yang et al., 2017). Moreover, optical transparency of the material should be considered to render the platform compatible with imaging applications.

## Organotypic Liver Models for Hepatotoxicity Predictions

### Scaffold-Free Spheroids in Multi-Well Formats

**Figure 2:**
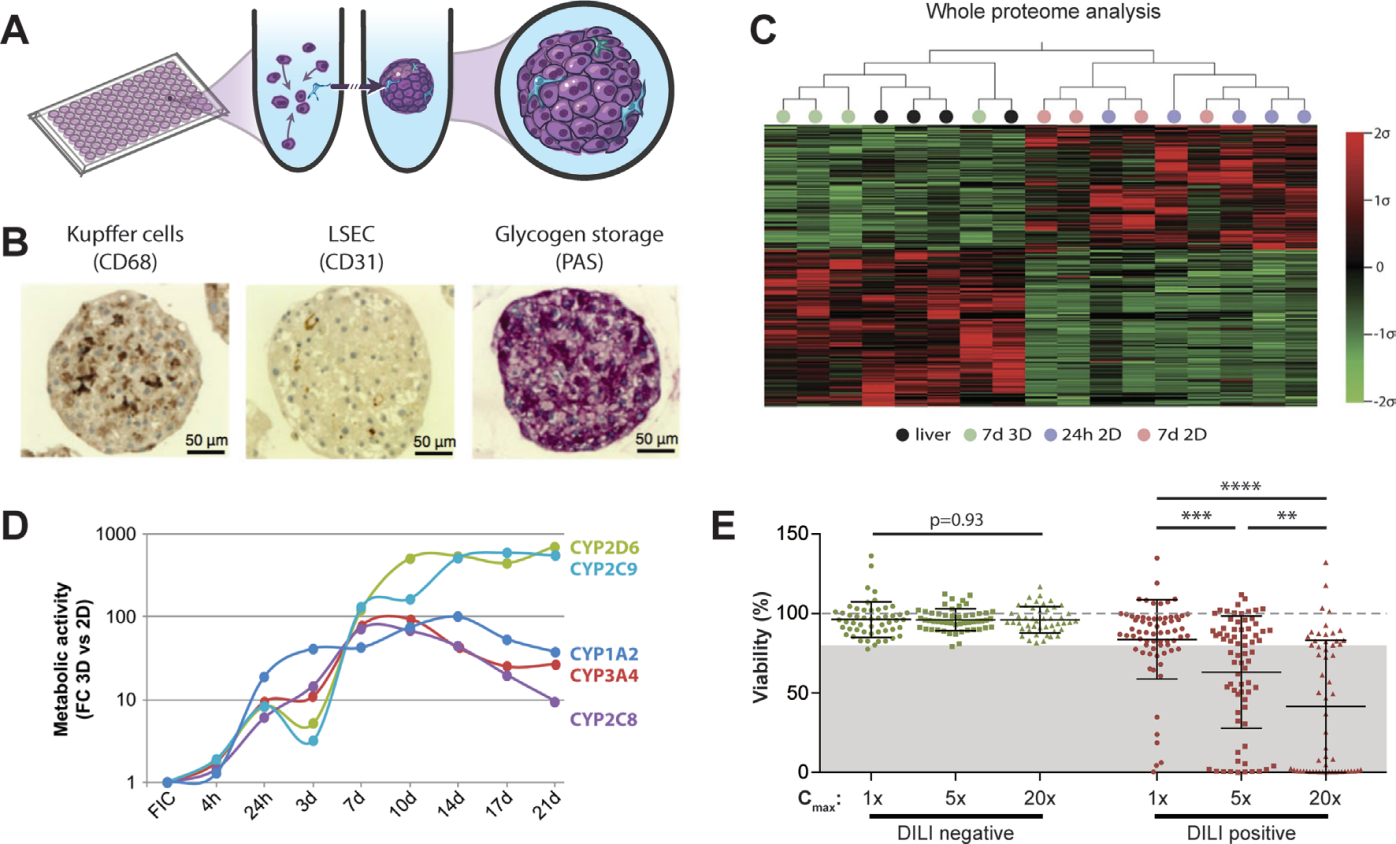
Primary human hepatocyte spheroids in chemically defined medium for DILI prediction. **(A)** Schematic representation of the spheroid formation process in ultra-low attachment plates. **(B)** Immunohistochemistry for CD68 and CD31 reveals presence of Kupffer cells and liver sinusoidal endothelial cells (LSEC). Periodic acid schiff (PAS) staining demonstrates glycogen storage capacity of PHH spheroids. **(C)** Mean-centered, sigma-normalized heatmap visualization of whole proteome data indicates that the proteomic signatures of PHH spheroids (7d 3D) closely resemble the proteomes of the corresponding human livers, whereas 574 proteins are differentially expressed in monolayer culture at different time points (24 h 2D and 7 days 2D). **(D)** Comparison of CYP metabolism determined by mass spectrometry between 2D and 3D spheroid cultures of the same donors (n = 3) demonstrates that metabolic activities are 10–900-fold higher in 3D PHH spheroids after one or more weeks in culture. FC: fold change; FIC: freshly isolated cells. **(E)** Toxicity assessment of 70 hepatotoxic drugs (DILI positive; red dots) and 53 DILI negative controls in PHH spheroids (green dots). Viability as determined by ATP quantifications relative to untreated controls is shown for three exposure levels (1×, 5×, and 20× therapeutic c_max_). The dashed line indicates viability of the respective control spheroids (100%). Notably, of the 70 compounds with DILI liabilities in humans, 48 of the 70 DILI positive compounds were successfully flagged as toxic (69% sensitivity), whereas none of the DILI negative drugs indicated hepatotoxic liabilities (100% specificity). Error bars indicate SD **, ***, and **** indicate p < 0.01, p < 0.001, and p < 0.0001 in a two-tailed heteroscedastic t-test, respectively. Figure modified with permission from (24, 58, 59; 61, 69).

Scaffold-free spheroids are 3D cell aggregates that form by self-aggregation of cells in suspension in the absence of added substrates that promote cell attachment (**Figure 2A**). Spheroids of controlled sizes can be formed over the course of multiple days, either in hanging drops or in multi-well plates with ultra-low attachment (ULA) surfaces. Additionally, spheroids can be formed on a large scale in stirred tank bioreactors, which we review in the section *Scaffold-Free Spheroids in Perfused Stirred-Tank Bioreactors*. The earliest reports of spheroid hepatocyte culture used newborn rat hepatocytes on poly-HEMA or proteoglycan-coated ULA surfaces and demonstrated that such spheroids were viable for multiple weeks and functionally superior compared to 2D monolayer culture (Landry et al., 1985; Koide et al., 1989; Tong et al., 1992). Building on these seminal findings, a plethora of studies established and characterized 3D spheroids using hepatoma cells, stem cell-derived hepatocyte-like cells (HLCs), or primary hepatocytes. In the following sections we will introduce different cell models for studies of liver function and drug metabolism and provide a comprehensive overview of their application for drug toxicity prediction.

#### Spheroids From Hepatic Cell Lines

Human liver cell lines, such as HepG2, HepaRG, and Huh-7, are commonly used for studies of drug metabolism and toxicity due to their low cost, unlimited availability, and vast available knowledge base. Interestingly, spheroid culture of hepatoma cells results in a down-regulation of ECM, cytoskeleton and adhesion molecules, cortical actin organization and increased activity of drug metabolizing enzymes, and enhanced liver-specific functions, such as apolipoprotein and albumin secretion (Chang and Hughes-Fulford, 2009; Takahashi et al., 2015).

HepG2 constitutes arguably the most extensively characterized hepatoma cell line. HepG2 spheroid culture resulted in polarized expression of MRP2 and MDR1 transporters, improved albumin secretion compared to HepG2 monolayer culture for several weeks, and increased susceptibility to acetaminophen-induced toxicity (TC_50_ of 7.2 mM in 3D culture compared to 33.8 mM in 2D culture) (Gaskell et al., 2016). Moreover, HepG2 spheroids are technically compatible with high-throughput screening (HTS) (Sirenko et al., 2016) and 3D culture improved prediction of hepatotoxicity of amiodarone, diclofenac, metformin, phenformin, and valproic acid (Fey and Wrzesinski, 2012). Importantly, however, the sensitivity to hepatotoxins in 3D spheroid culture was still considerably lower than in 3D PHH culture or *in vivo*, in accordance with low expression and activity of drug metabolizing enzymes (Wilkening et al., 2003). Of note, as cancer-derived cells, HepG2 models were found to be sensitive to compounds with anti-proliferative effects that do not rely on hepatic metabolism (Sirenko et al., 2016).

Compared to HepG2, HepaRG cells exhibit higher metabolic activity (Hart et al., 2010; Nelson et al., 2017; Yokoyama et al., 2018). HepaRG spheroids can be cultured up to 7 weeks with polarized transporters and bile canalicular structures (Leite et al., 2012). Furthermore, activities of CYP1A2, CYP2B6, CYP2C9, CYP3A4, and UGT enzymes remained stable and substantially (2- to 20-fold) higher in 3D compared to 2D culture (Leite et al., 2012; Wang et al., 2015; Ott et al., 2017). In accordance with these functional data spheroid culture improved sensitivity to acetaminophen and aflatoxin B1, which both require metabolic activation (Leite et al., 2012; Gunness et al., 2013; Mueller et al., 2014). HepaRG spheroids furthermore distinguished trovafloxacin and troglitazone from their less toxic structural analogues (Ramaiahgari et al., 2014). In a small set 10 hepatotoxins and 2 non-toxins using a multiplexed HepaRG spheroid assay, the platform correctly flagged 7/10 compounds as hepatotoxic after exposure for 7 days, whereas 6/10 drugs were successfully identified in the corresponding monolayer culture (Ott et al., 2017). Effects of the culture paradigm on drug sensitivity appears to be compound-specific, as 2D HepaRG monolayer culture was the more sensitive model for the detection of troglitazone, tamoxifen, and chlorpromazine toxicity (Gunness et al., 2013; Mueller et al., 2014; Ott et al., 2017).

In addition to being used as a model for hepatocellular toxicity, Leite and colleagues recently demonstrated that HepaRG-stellate cell co-cultures can be used to detect stellate cell activation and drug-induced liver fibrosis of allyl alcohol and methotrexate (Leite et al., 2016). Furthermore, the authors found that acetaminophen could activate stellate cells, which was confirmed *in vivo* in mice, thus providing a useful experimental tool for the prediction of drug-induced liver fibrosis.

#### Spheroids From Primary Human Liver Cells

PHH serve as the gold standard cell type for predicting human hepatotoxic drugs (Gómez-Lechón et al., 2014). However, their rapid dedifferentiation in conventional 2D monolayer culture limited their phenotypic advantages compared to cell lines to short-term studies. Strikingly, organotypic spheroid culture provided a major conceptual advancement. In spheroids, PHH remain viable with stable albumin secretion for at least 5 weeks (Messner et al., 2013; Bell et al., 2016). Moreover, they can be co-cultured with NPCs and are capable of glycogen storage (**Figure 2B**). Importantly, we could show that proteomic signatures in 3D spheroids closely resemble the human liver *in vivo*, whereas cultures from the same donors rapidly deteriorated in 2D monolayer or sandwich culture (**Figure 2C**). Similarly, PHH spheroids retained their transcriptomic and metabolomic profiles for multiple weeks (Bell et al., 2016; Bell et al., 2017; Vorrink et al., 2017; Bell et al., 2018). In contrast, transcriptomic patterns of other emerging cell models differed substantially and 8,148 out of 17,462 genes analyzed were differentially expressed in PHH spheroids compared to HepaRG cells and stem cell-derived HLCs (Bell et al., 2017). In accordance with these phenotypic differences, PHH spheroids exhibited substantially increased activity of CYP1A2, CYP2B6, CYP2C8, CYP2C9, CYP2C19, CYP2D6, and CYP3A4 compared to HepG2, HepaRG, and 2D cultured PHH (**Figure 2D** and references Berger et al., 2016; Vorrink et al., 2017).

These phenotypic improvements over other cell models and culture paradigms rendered PHH spheroids a promising tool for hepatotoxicity studies. Ogihara et al. demonstrated that PHH spheroids could detect hepatotoxicity of compounds with diverse toxicity mechanisms, such as acetaminophen, chlorpromazine, diclofenac, flutamide, imipramine, ticlopidine, and troglitazone, at clinically relevant concentrations after 2 weeks exposure using aspartate aminotransferase (AST) leakage as readout (Ogihara et al., 2015; Ogihara et al., 2017). In addition, spheroid assays have been presented that are tailored to identify and study specific hepatotoxicity mechanisms, such as cholestasis (Hendriks et al., 2016) and steatosis (Kozyra et al., 2018).

Recently, two studies evaluated the predictive power of PHH spheroids offered by InSphero AG in media with proprietary composition to detect hepatic liabilities of selected compounds. Proctor et al. analyzed 110 drugs (69 DILI positive and 41 DILI negative) at concentrations up to 100× the therapeutic exposure levels for 2 weeks in undisclosed media formulations and reported sensitivity and specificity of 59% and 80%, respectively (Proctor et al., 2017). The same model was compared to 2D sandwich culture using 12 DILI positive test compounds (Richert et al., 2016). Surprisingly, the authors found that 2D sandwich culture correctly identified 11/12 DILI-positive compounds already after 3 days, whereas the spheroid model only detected 9/12 compounds after 14 days (amiodarone, bosentan, and ximelagatran as false negatives).

In contrast to the InSphero model, we used spheroids in chemically defined serum-free conditions (CD-spheroids) to evaluate the toxicity of 123 drugs (70 DILI positive and 53 DILI negative) (Vorrink et al., 2018). Of these drugs, 38 overlapped with the study by Proctor and colleagues. The model achieved 69% sensitivity and 100% specificity at exposure levels of 20× therapeutic c_max_ (**Figure 2E**). Moreover, CD-spheroids correctly flagged amiodarone and bosentan as hepatotoxins. Combined, these results emphasize that PHH spheroids provide accurate tools for the preclinical prediction of hepatic liabilities of compounds with diverse patterns of liver damage from various therapeutic areas.

#### Spheroids From Stem Cell-Derived Hepatocyte-Like Cells

HLCs can be obtained by differentiation from human induced pluripotent stem cells (hiPS-Hep) and have been discussed as a promising alternative *in vitro* model for studies of human liver function and drug toxicity. To date, various differentiation protocols have been presented. While the overall differentiation state of HLCs has been improved, current protocols still fall short from producing cells that closely resemble the molecular phenotype of mature PHH (Schwartz et al., 2014; Baxter et al., 2015; Goldring et al., 2017). Importantly, however, while their phenotype remains immature, HLCs offer the appealing advantage that they can be generated from material from patients with specific phenotypes of interest, for instance from those that experienced rare idiosyncratic drug reactions, thus providing the unique opportunity to investigate patient-specific risk factors.

As for other cell models, spheroid culture seems to improve hepatic phenotypes and support expression of marker genes, albeit the reached levels are still orders of magnitude lower than in PHH (Meier et al., 2017). Despite these beneficial effects, only few studies have used HLC spheroids for hepatotoxicity assessments. Takayama and colleagues cultured HLC spheroids in multi-well plates with nanopillars at the bottom of each hole to facilitate spheroid formation (Takayama et al., 2013). Notably, in this platform, HLC spheroids were overall more sensitive than HepG2 spheroids to a set of 24 hepatotoxins. However, sensitivity was considerably lower than PHH monolayer cultures. By contrast, Sirenko *et al*. found that HepG2 spheroids were much more sensitive than HLC spheroids for 10/23 evaluated compounds following a single 72-h exposure (Sirenko et al., 2016). In summary, while 3D culture seems to improve hepatic phenotypes, its effects on sensitivity to hepatotoxins remain to be determined.

#### Spheroids From Primary Animal Hepatocytes

Organotypic liver models using animal cells can have multiple applications. Most commonly, primary animal hepatocytes, particularly from rat, are used to study human drug response, providing a cheaper and more easily accessible cell source. As for PHH, these spheroids possess structural polarity and functional bile canaliculi with MDR1 and MRP2 being localized at the canalicular membranes (Kyffin et al., 2019). Furthermore, rat hepatocyte spheroids retain the expression of hepatic genes, including albumin drug metabolizing enzymes and clotting factors for multiple weeks (Abu-Absi et al., 2002; Brophy et al., 2009). Exposure of rat hepatocyte spheroids to acetaminophen decreased glutathione levels and intracellular ATP, however at concentrations vastly exceeding toxic levels in humans (Sanoh et al., 2014). This relative insensitivity might be due to the drastic downregulation of Cyp3a2 and Cyp2e1, orthologues of human CYP3A4 and CYP2E1 that are mediating the formation of the liver toxic reactive acetaminophen metabolite NAPQI. Rat spheroids have furthermore been used to test the hepatotoxicity of methotrexate. Interestingly, the authors found that spheroids were less sensitive to methotrexate hepatotoxicity than 2D monolayer cultures, likely due to increased MRP2-mediated export (Walker et al., 2000; Yin et al., 2009).

Various strategies have been proposed to use animal hepatocytes as proxies for specific human genotypes. For instance, as cats lack UGT1A6 and have reduced activity of UGT2B isoforms (Court, 2013), cat hepatocytes could be useful to model the reduced glucuronidation capacity in individuals with poor UGT activity, for instance for mimicking the increased susceptibility to acetaminophen hepatotoxicity in individuals with Gilbert’s syndrome (de Morais et al., 1992). Similarly, as dogs lack the *N*-acetyltransferases *NAT1* and *NAT2* (Trepanier et al., 1997), dog hepatocytes could model the effects of decreased NAT activity, which is common in humans (up to 40% are slow NAT metabolizers). Importantly, however, while such experiments might inform about the contribution of specific metabolic pathways, drastic differences in overall isoform composition, expression, and catalytic activities of drug metabolizing enzymes limit the predictive power of organotypic animal liver models for the prediction of human hepatotoxicity (Martignoni et al., 2006).

### Scaffold-Free Spheroids in Perfused Stirred-Tank Bioreactors

Perfused stirred-tank bioreactors feature stirring to promote cellular aggregation. Similar to scaffold-free spheroids in multi-well formats, spheroids in bioreactors displayed spontaneous assembly of functional bile canaliculi networks, physiologically relevant expression of hepatocyte-specific markers, and sustained CYP activity (Tostões et al., 2012). Furthermore, the model has been expanded to be co-cultured with an outer layer of stromal human bone marrow mesenchymal stem cells, and the authors report increased expression of CYP3A4 and CYP1A2 (Rebelo et al., 2017).

Bioreactors allow tight control of parameters such as dissolved oxygen and pH and enable spheroid formation at scales that are difficult to achieve in conventional multi-well plate formats. Key parameters to consider are the type and size of paddle and impeller as well as the rate of mixing in order to balance the establishment and maintenance of cell–cell contacts with sheer stress. Furthermore, it must also be ensured that autocrine and paracrine signals are not disrupted by high perfusion rates (Ebrahimkhani et al., 2014).

Importantly, perfused stirred-tank bioreactors do not allow the testing of different conditions without transfer to separate compartments, as spheroids are formed in a single reaction chamber. As such, the main application of these systems is to feed multiplexed high-throughput platforms for hepatotoxicity testing or other applications. Problematic in this context, however, is the large variability in spheroid sizes that can negatively impact reproducibility and test standardization.

### Scaffold-Based Culture Models

Organotypic liver cultures can be supported by a variety of scaffolds, i.e., natural or synthetic components that can facilitate the formation and maintenance of cell–cell contacts, cell polarity, and tissue organization. In recent years, particularly alginate, collagen and nanofibrous poly-L-lactic acid (PLLA) scaffolds have received growing attention in the context of hepatic spheroids, as they facilitate the deposition of essential components of the hepatic microenvironment, including collagens and fibronectin (Selden et al., 2000). Alginate microencapsulation consequently improves viability and metabolic capacity of HepG2 and HepaRG spheroids (Elkayam et al., 2006; Rebelo et al., 2015). Alginate and alginate-collagen composite hydrogel furthermore enhance hepatic functions of primary rat hepatocyte spheroids formed using high-throughput emulsion droplet microfluidics (Chan et al., 2016). Similarly, PLLA nanoscaffolds had beneficial effects on albumin secretion and slightly improved expression of Cyp1a2 and Cyp2b2 of primary rat hepatocytes after 7 days in culture compared to 2D monolayers (Bierwolf et al., 2011). Also for PHH, beneficial scaffold effects on hepatic function have been demonstrated, manifesting in improved clearance predictions of low clearance compounds, such as warfarin and coumarin (Phillips et al., 2018).

Only few studies have analyzed the effects of scaffolds in the context of hepatotoxicity assessments. Spheroid culture on polystyrene scaffolds increased the activity of Cyp1a2, Cyp2b1, and Cyp3a2 more than fourfold and improved the sensitivity of rat hepatocyte spheroids to acetaminophen (Schutte et al., 2011). TC_50_ values (approximately 40 mM), however, were still substantially higher than in scaffold-free spheroid culture or *in vivo* (approximately 1 mM). In contrast, HepG2 spheroids in ECM-based hydrogel ceased to proliferate and acquired a more differentiated phenotype, indicated by gradually increased expression of albumin, hepatic transcription factors, phase I and II drug metabolism enzymes, and transporters up to day 28, whereas culture in sandwich configuration or on porous polystyrene scaffold did not result in phenotypic improvements (Ramaiahgari et al., 2014; Luckert et al., 2017). Consequently, HepG2 spheroids in Matrigel identified the toxicity of eight hepatotoxins, whereas four control compounds not implicated in DILI were correctly flagged as nontoxic (Ramaiahgari et al., 2014).

Liu et al. established HepaRG spheroids using decellularized rat liver as scaffold. They showed that this liver biomatrix scaffold enhances expression of phase I and phase II enzymes, drug transporters, and nuclear receptors for up to 28 days compared to scaffold-free spheroid culture (Liu et al., 2018). However, expression levels of most genes were still considerably lower than in PHH. In accordance with these improved molecular phenotypes, toxicity testing of 14 hepatotoxins revealed that the addition of scaffolds conferred a higher DILI sensitivity. Furthermore, various other culture scaffolds, such as galactosylated cellulosic sponges or poly-ethylene-glycol-diacrylate, increased long-term hepatic functionality of spheroids generated from human liver cell lines or stem cell-derived hepatocytes (Khalil et al., 2001; Tasnim et al., 2016; Wang et al., 2016; Mun et al., 2019). Furthermore, hepatotoxicity tests using small panels of drugs indicated that these models can successfully identify the hepatotoxicity of selected drugs *in vitro*.

While the highlighted studies indicate beneficial effects of various scaffold materials on the phenotypes of hepatic spheroids, comprehensive benchmarking experiments in which the molecular signatures of scaffold-based hepatic cultures have been compared on transcriptomic, proteomic, and metabolomic levels to the intact liver have not been conducted. Furthermore, batch-to-batch variability of scaffold materials can impair reproducibility and comparability, thus complicating the application of these models for DILI predictions. Additionally, the effects of scaffolds on drug diffusion should be considered and exposure concentrations should be adjusted accordingly before applying scaffold-based models to pharmacological and toxicological analyses.

### Micropatterned Co-Culture

**Figure 3:**
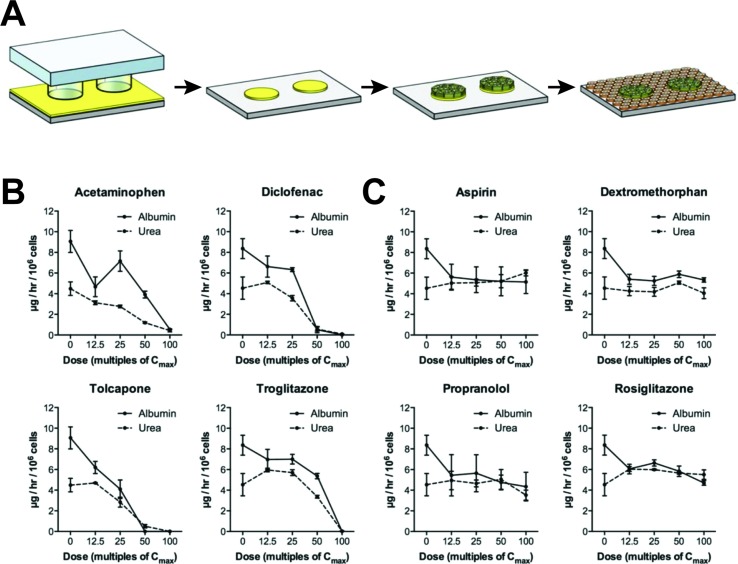
Micropatterned co-cultures (MPCC) support phenotypic maintenance of primary human hepatocytes and hiPS-Heps. **(A)** MPCCs are established by seeding PHH or hiPS-Heps on micropatterned islands of ECM substrate, followed by seeding of supportive murine fibroblasts. Albumin and urea secretion as proxies for cell health can distinguish four hepatotoxins **(B)** from four nontoxic compounds **(C)** after 8 days of exposure in hiPS-Hep MPCC. Figure modified with permission from (Berger et al., 2015).

As an alternative to spheroid culture, Bhatia and colleagues developed a micropatterned system in which hepatic cells are seeded on micropatterned ECM islands and are supported by surrounding murine fibroblasts (**Figure 3A**). PHH in such MPCC form bile canaliculi networks and retain expression of phase I and phase II drug metabolizing enzymes, nuclear receptors, as well as drug transporters for at least 6 weeks in culture (Khetani and Bhatia, 2008). MPCC are widely used and have been shown to be a valuable tool for different phases of drug development by closely mimicking the complexity of human liver (Ware and Khetani, 2017).

For hepatotoxicity tests, the model achieved 65% sensitivity and 90% specificity when analyzing a panel of 45 drugs (35 DILI positive and 10 DILI negative) using glutathione and ATP levels, as well as albumin secretion and urea synthesis as endpoints (Khetani et al., 2013). In addition to PHH, MPCC has been used to culture HLCs derived from iPSCs (iMPCC) overlaid with a thin Matrigel layer (Berger et al., 2015). The majority of hepatic genes in iMPCCs were expressed at levels between 10% and 200% of suspension cultured hepatocytes. Moreover, global gene expression profiling revealed that iMPCC transcriptomic signatures remained relatively stable between day 9 and day 21 of culture (R^2^ between 0.6 and 0.96). Functional parameters, such as the activities of CYP2A6 and CYP3A4, as well as glucuronidation and sulfation reactions were drastically decreased to 5–25% of PHH levels. The system successfully distinguished between four toxic and four nontoxic compounds (**Figures 3B, C**) and later extension to a panel of 47 drugs yielded 65% sensitivity and 100% specificity, which was similar to the result in the PHH MPCC system on the same panel of test drugs, thus corroborating the value of iMPCC for DILI predictions (Ware et al., 2015).

### Hollow Fiber Bioreactors

**Figure 4:**
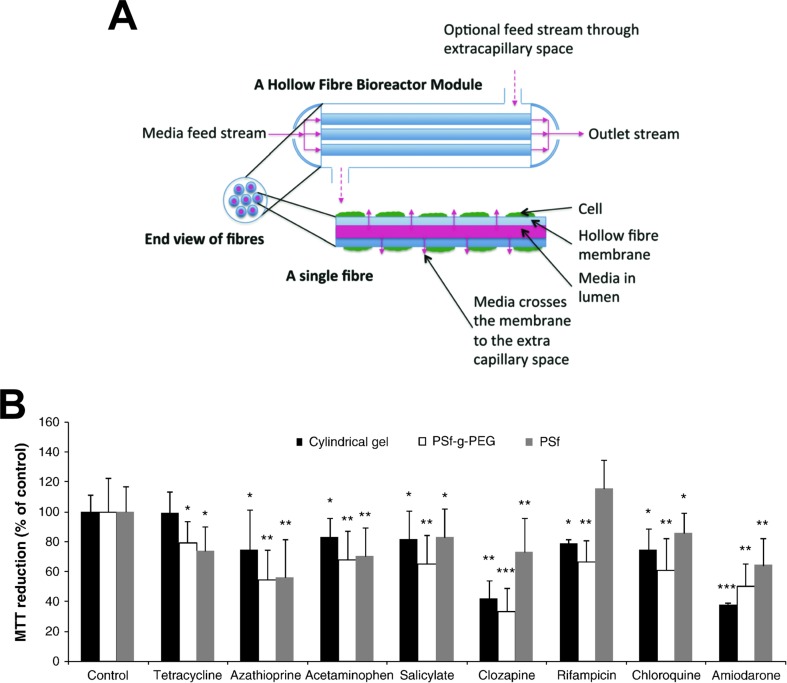
Hollow fiber bioreactors for organotypic hepatocyte culture. **(A)** Schematic representation of a hollow fiber bioreactor setup. Perfusion capillaries pass through a bioreactor module supplying the cells in the extracapillary space with oxygen and nutrients. **(B)** Hepatotoxicity of eight drugs was evaluated in bioreactors with hollow fibers made from polysulfone-g-poly(ethylene glycol) (PSf-g-PEG) or polysulfone (PSf) and was compared to cultures in cylindrical gels without hollow fibers. PSf-g-PEG fibers drastically reduced protein absorption and supported cellular functions as well as drug sensitivity. Error bars indicate ± SD; *p < 0.05, **p < 0.01, and ***p < 0.001. Figure modified with permission from (Shen et al., 2010; Williams et al., 2013).

In hollow fiber bioreactors, cells are cultured in tubular modules, each permeated by a network of intratubular capillaries, which allow perfusion of medium and gases (**Figure 4A**). Hepatic cells are cultured in the surrounding extra-capillary space, which permits the mimicking of capillary blood-tissue exchange, thus providing a pseudovascularized tissue model in which cells are protected from direct media flow (Williams et al., 2013).

Hollow fiber bioreactors for artificial liver support developed by Gerlach and colleagues require around 10^10^ cells in a cell compartment of 800-ml volume, which renders such approaches incompatible with parallel drug testing (Gerlach et al., 1994; Gerlach et al., 2003; Sauer et al., 2003; Pless et al., 2006). In an attempt to make this setup accessible for pharmacological studies, the bioreactor setup was miniaturized to cell compartment volumes of 0.5 to 2 ml accommodating 10^7^ to 10^8^ cells. Notably, miniaturization did not negatively impact hepatic functions and metabolic activities were preserved for multiple weeks (Zeilinger et al., 2011; Hoffmann et al., 2012). Transporter expression of *SLCO1B1* (OATP1B1) in HepaRG bioreactor cultures was 95% lower than in PHH bioreactors, whereas *CYP3A4* levels were found to be similar (Ulvestad et al., 2012). While all major *in vivo* metabolites of diclofenac were detected in both PHH and HepaRG bioreactors, metabolic routes of the investigational peroxisome proliferator activated receptor alpha (PPARA) agonist AZD6610 differed in HepaRG bioreactors, whereas they were similar between PHH and *in vivo* (Darnell et al., 2012). Furthermore, Lübberstedt et al. demonstrated that serum-free culture of PHH in the miniaturized bioreactor resulted in increased expression of various CYPs, phase II enzymes, and drug transporters compared to cultures maintained with fetal calf serum (Lübberstedt et al., 2015).

To further facilitate mass exchange of nutrients and metabolites between cells and culture medium, de Bartolo and colleagues arranged the fibers of their bioreactor in cell plates with bilateral sinusoidal structures (De Bartolo et al., 2009). The bioreactor features two types of alternating cross-assembled hollow fiber membranes with different molecular weight cut-offs and physicochemical properties. These differing membrane properties allow the two membrane systems to mimic the *in vivo* arterious and venous blood vessels. When cryopreserved PHH were cultured in the system, urea synthesis and albumin production were maintained at levels approximately 8-fold higher than in the liver support system and diazepam biotransformation was observable for 18 days.

Due to the large cell requirements, hollow fiber bioreactors have rarely been used for hepatotoxicity evaluations. To our knowledge, only two studies have been presented for the utility of hollow fiber bioreactors for hepatotoxicity evaluations, both using fresh rat hepatocytes. In their first study, Shen and colleagues demonstrated that bioreactor cultures of rat hepatocytes were more sensitive than the corresponding 2D cultures for the detection of acetaminophen hepatotoxicity, likely due to higher CYP2E1 activity (Shen et al., 2006). However, as rats are highly resistant to acetaminophen toxicity *in vivo* (McGill et al., 2012), the relevance of these results can be questioned. In a follow-up work, the same group tested 48-h exposures to eight drugs (**Figure 4B**). The main findings were that rat hepatocytes were more sensitive when cultured in polysulfone-g-poly(ethylene glycol) hollow fiber bioreactors compared to those cultured either in bioreactors with polysulfone hollow fibers or without hollow fibers (Shen et al., 2010).

In summary, hollow fiber bioreactors provide an excellent culture system for the long-term maintenance of hepatic phenotypes and functions. However, they are expensive, difficult to establish, and even in the miniaturized setup bioreactors require cell numbers that are orders of magnitude higher than in other organotypic 3D culture paradigms (Planchamp et al., 2003). Furthermore, toxicological and pharmacological studies in hollow fiber bioreactors are complicated by the absorption of hydrophobic drugs by system components and limited accessibility to biochemical or imaging-based monitoring of functional parameters.

### Perfused Liver Chips

**Figure 5:**
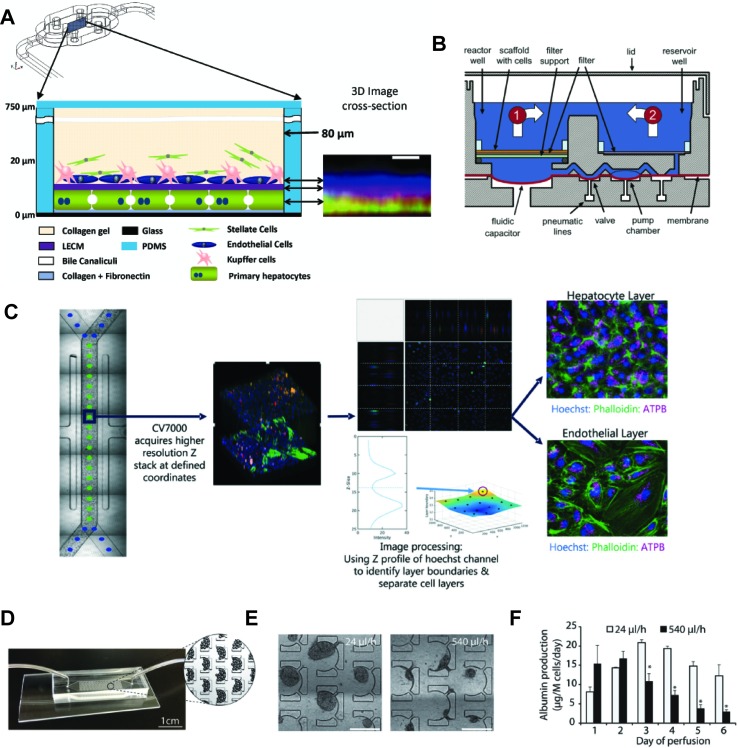
Selection of different chip models for the culture of hepatic cells. **(A)** Design and layering schematic of the Liver Acinus MicroPhysiology System (LAMPS) developed by Taylor and colleagues. The system features a layered architecture in which PHH are sandwiched between layers of ECM and overlaid with Kupffer, stellate, and endothelial cells (from dermal isolates) in a collagen gel scaffold. Inlet on the right shows reconstruction of the liver acinus from confocal images. Scale bar = 10 µm. **(B)** Schematic cross-section of one fluidically isolated reactor compartment from the LiverChip developed by Griffith and colleagues. Cells in compartment one are continually perfused through the scaffold by a diaphragm micropump, which circulates medium between the two wells. **(C)** Automated imaging workflow for the Emulate liver chip. The CV7000 automated microscope first performs low-resolution bright field scans of up to eight chips. On the basis of these images, relevant fields of view can be determined, which are subsequently used to high-resolution Z-stack imaging that allows accurate separation of the hepatocyte and endothelial cell layers. **(D–F)** Platform for the perfusion of hiPS-Hep spheroids developed by Bhatia and colleagues. **(E)** PDMS chips were designed with C-shaped features of 500 µm that allow to entrap spheroids across a range of perfusion rates. **(F)** Albumin secretion could be detected after 1 week of perfusion with flow rates up to 540 µl/h. Error bars indicate SEM; *p ≤ 0.05. Figure modified with permission from (Domansky et al., 2010; Schepers et al., 2016; Lee-Montiel et al., 2017; Peel et al., 2019).

Liver cells can be cultured on microfluidic “chip-like” devices, which incorporate media perfusion to improve nutrient supply and mimic physiological shear stress (Cui and Wang, 2019). These liver chips typically feature a layered architecture in which layers of hepatocytes and NPCs are cultured adjacently, separated by ECM deposits (**Figure 5A**). Importantly, molecular phenotype and function of hepatocytes differ along the lobular axis; levels of urea synthesis, gluconeogenesis, and beta-oxidation are highest in the oxygen-rich periportal zone (approx. 15% oxygen) and decrease towards the oxygen-poor region around the central vein (approx. 6% oxygen), whereas glycolysis, bile acid synthesis, and CYP metabolism show inverse profiles (Kietzmann, 2017). Importantly, perfusion can be used to recapitulate this complex zonal architecture of the liver, resulting in graded expression of zone-specific genes, such as CYP3A4, CYP2E1, A1AT, and Arginase 1, and recapitulation of the perivenous pattern of acetaminophen hepatotoxicity (Lee-Montiel et al., 2017; Tomlinson et al., 2019).

The LiverChip manufactured by CN Bio consists of multiple units of collagen-coated reactor compartments in which hepatocytes assemble into 3D microtissues, as well as medium reservoirs and integrated micropumps (**Figure 5B** and Powers et al., 2002; Domansky et al., 2010). PHH in the LiverChip form bile canaliculi and exhibit detectable levels of CYP activity. However, expression levels of various CYPs (CYP2C8 and CYP2E1), drug transporters (BSEP and OATP1B3), and nuclear receptors (CAR and PXR) decline considerably over the course of 10 days (Vivares et al., 2014). Nevertheless, hierarchical clustering of lactate dehydrogenase (LDH) leakage, albumin secretion and urea production distinguished the hepatotoxins fialuridine, acetaminophen, and clozapine from the nontoxins olanzapine and entecavir (Rowe et al., 2018). In a co-culture configuration of PHH and Kupffer cells, the platform remained metabolically stable for 2 weeks and could model the modulation of CYP3A4 activity by the monoclonal antibody tocilizumab and its effects on simvastatin hydroxy acid pharmacokinetics (Long et al., 2016). Interestingly, the platform has furthermore proven useful for the analysis of interactions of the hepatic niche with infiltrating breast cancer cells (Wheeler et al., 2014; Clark et al., 2017).

The HµREL chip is a polycarbonate microdevice that consists of multiple microfluidically interconnected cell compartments, fluid reservoirs, and peristaltic pumps. This system has been successfully used for the relatively accurate prediction of drug clearance in man (Chao et al., 2009; Novik et al., 2010). Interestingly, flow seemed to improve CYP1A2, CYP2B6, and CYP3A4 activity only when PHH were co-cultured with NPCs (Novik et al., 2010). It can be hypothesized that an explanation for these findings is the secretion of collagen by stellate cells, as collagen has been demonstrated to be essential for the enhanced functional response of hepatocytes cultured under flow (Hegde et al., 2014). Furthermore, the model was recently used to screen 19 compounds (12 DILI positive, 7 DILI negative) with known clinical hepatotoxic liabilities (Novik et al., 2017). Using 100× human c_max_ as threshold, the authors successfully flagged 10 out of 12 compounds as hepatotoxic. However, sensitivity was substantially lower (i.e., TC_50_ values were substantially higher) than in spheroids and MPCC (compare (Khetani et al., 2013; Vorrink et al., 2018).

Emulate Bio has recently presented a PDMS-based chip consisting of two parallel channels coated with a proprietary ECM mixture and separated by a porous membrane. The upper channel contains monolayers of hepatocytes while the lower channel features liver sinusoidal endothelial cells (LSEC) (Peel et al., 2019). Notably, the chip layout is standardized and compatible with automated microscopy to enable high-throughput evaluation of imaging-based endpoints (**Figure 5C**). Hepatotoxicity assessment of acetaminophen and fialuridine resulted in dose- and time-dependent increases in miR-122 and α-GST release as well as decreases in albumin secretion (Foster et al., 2019). Notably though, hepatotoxicity of fialuridine (TC_50_ in chip: 84 µM; TC_50_ in sandwich culture: 45–90 µM) and acetaminophen (TC_50_ in chip: 2.4 mM; TC_50_ in sandwich culture: 2–3 mM) was similar to previous reports in sandwich cultured PHH (Bell et al., 2018), and thus, the added value of perfusion and LSEC co-culture for DILI prediction is not immediately clear.

Draper recently presented a liver-on-a-chip system made of oxygen permeable thermoplastic that consists of 96 units, each containing sandwich cultured hepatocytes maintained in recirculating microfluidic conditions (Tan et al., 2019). The authors demonstrate that flow substantially increases albumin secretion and CYP3A4 activity but did not yet present the platform’s performance for pharmacological or toxicological applications. Mimetas provides microfluidic plates consisting of culture chambers with three juxtaposed inlets and outlets in which perfusion is generated by gravity on a rocking device. While this platform has been extensively used for renal and neural cultures, only a single proof-of-concept study has been presented to date, which indicated that hepatic cells (HepaRG) remained largely viable and polarized for 14 days (Jang et al., 2019).

An interesting device was developed by Schepers and colleagues in which spheroidal hiPS-Hep aggregates are entrapped in perfusable C-shaped features (**Figures 5D–F**). Cells in this model remain viable and secrete albumin for at least 38 days, and the platform tolerates large variations in perfusion rate (from 24 up to 500 µl/h) (Schepers et al., 2016). Jin and colleagues presented a microfluidic device featuring rocker-based gravity-driven perfusion and vascularized liver organoids (Jin et al., 2018). Organoids were formed from HLCs generated by direct transcription factor-mediated reprogramming of murine fibroblasts. Notably, co-culture with HUVECs and culture in a decellularized porcine liver ECM hydrogel improved expression profiles, as well as albumin secretion, urea production, and CYP3A4 activity of these induced hepatic cells compared to monocultures without supportive ECM scaffold. Furthermore, the model exhibited dose-dependent hepatotoxicity of ethanol and acetaminophen, albeit at concentrations >10-fold higher than clinically toxic levels. Furthermore, the authors present proof-of-concept data showing the integration of the system with gastric and small intestinal organoids for pharmacokinetic studies.

In addition, a plethora of other liver chips have been presented. However, these models are only in their infancy and no comprehensive characterization data have been presented with regards to molecular hepatic phenotypes or predictive power for DILI predictions. For further details on hepatic chip platforms, we refer the interested reader to recent reviews on the topic (Beckwitt et al., 2018; Cui and Wang, 2019).

### Bioprinting

3D bioprinting encompasses a variety of methodologically distinct modalities that can create complex biological tissue structures by depositing cells and extracellular factors with high spatial and temporal precision. Droplet-based bioprinting (DBB), also termed inkjet bioprinting, refers to the precise deposition of cells dispensed in small droplets (Gudapati et al., 2016). Advantages of this method are high resolution down to 2 µm for hydrogels and down to 50 μm for cells and the high throughput that allows printing of cm-sized tissue constructs (Kang et al., 2016). For liver cells, DBB has been used to produce heterocellular systems of hepatic (HepG2) and endothelial (HUVEC) cell lines in which albumin secretion and CYP3A4 activity were increased up to threefold compared to HepG2 monocultures (Matsusaki et al., 2013). Furthermore, this modality does not impair the pluripotency of embryonic or iPSCs and is compatible with post-printing differentiation into HLCs (Faulkner-Jones et al., 2015).

Extrusion-based bioprinting (EBB) results in the deposition of continuous filaments of desired shapes, differing from the individual droplets of inkjet-based bioprinters, and constitutes arguably the most widely used bioprinting modality (Ozbolat and Hospodiuk). This method is highly versatile and compatible with a wide range of bioink properties. Furthermore, it currently constitutes the only modality compatible with the printing of scaffold-free cell systems, such as spheroids (Skardal et al., 2015;Bhise et al., 2016; Kizawa et al., 2017). However, EBB is rather slow and can result in considerable shear stress during the printing process. Furthermore, resolution is more limited than in other bioprinting modalities (>100 µm), rendering EBB incompatible with printing capillary networks. In the context of liver cells, EBB has been successfully used for a variety of applications in pharmacology, toxicology, and regenerative medicine. Multilayered 3D structures of HepG2 cells using alginate scaffolds expressed detectable levels of hepatic markers, such as ALB, TAT, and ASGR1 (Jeon et al., 2017). Yet, increased expression levels of the fetal liver marker AFP indicate that mature hepatic phenotypes are not achieved in this model.

Notably, EBB also has important prospects for tissue engineering. In an effort to incorporate vascularization, Lee et al. (2016) printed primary rat hepatocytes, endothelial HUVECs, and human lung fibroblasts in collagen bioink and demonstrated that the heterotypic interactions with NPCs improved albumin secretion and urea synthesis by >20-fold over the course of 10 days compared to hepatocyte monocultures. Furthermore, HLCs differentiated from mouse iPSCs and bioprinted using EBB into 3D hepatic structures further matured *in vivo* when transplanted into recipient mice in a liver injury model, suggesting the utility of such approaches for regenerative medicine applications (Kang et al., 2018).

Light-assisted bioprinting (LAB) constitutes the least prevalent bioprinting modality, primarily due to its high cost, difficult handling, and lack of commercially available off-the-shelf solutions. However, due to its high resolution and printing speed coupled with low cell stress during the printing process, LAB constitutes a promising platform for tissue engineering. LAB allows bioprinting of structures mimicking the microscale hexagonal architecture of the human liver in which cells display improved molecular marker signatures compared to conventional 2D monolayer culture (Grix et al., 2018). Furthermore, co-culture of hiPS-Heps in this system together with HUVEC and adipose-derived stem cells resulted in improved hepatic maturation for at least 1 week following bioprinting (Ma et al., 2016).

Bioink is a key component of the bioprinting process that should offer mechanical support for the printed structure while mimicking the natural microenvironment of the encapsulated cells. Recent years have seen tremendous progress in bioink development (Gopinathan and Noh, 2018; Gungor-Ozkerim et al., 2018). For hepatic cells, however, only alginate, Matrigel, gelatin methacrylamide, and decellularized ECM-based bioinks have been employed to date. Alginate is used for its similarity to glycosaminoglycans of human ECM and is fully biocompatible (Axpe and Oyen, 2016). Furthermore, alginate has beneficial gelation properties and is relatively cheap. Alginate encapsulated HepG2 cells exhibited higher urea synthesis compared to 2D monolayer cultures despite reduced cell viability caused by dispensing pressure and nozzle diameter (Chang et al., 2008; Hwang et al., 2010). However, when alginate cellulose nanocrystal hybrid bioink was used for printing of human hepatoma cells in 3D structures resembling liver sinusoids, only minor effects of the encapsulation and printing process on cell viability were observed (Wu et al., 2018).

Promising data have been presented for DILI tests using bioprinted liver models. Specifically, bioprinted livers reproduced hepatotoxicity of acetaminophen at clinically relevant concentrations and successfully distinguished trovafloxacin from its nontoxic structural analogue levofloxacin (Knowlton and Tasoglu, 2016; Nguyen et al., 2016). Furthermore, bioprinted liver has successfully been used for mimicking TGF-β1 and methotrexate induced fibrinogenesis (Norona et al., 2019). Combined, these studies indicate that bioprinting constitutes an emerging technology that supports the maintenance of hepatic phenotypes and functionality. Furthermore, the possibility to precisely mimic the architecture of the intact organ *in vitro* opens a multitude of promising liver-related applications. However, while promising proof-of-concept data have been presented, the added value for the prediction of drug metabolism and toxicity in bioprinted liver models compared to other 3D culture models has not yet been demonstrated.

### Critical Comparison of DILI Prediction Models

While a large number of studies using organotypic and microphysiological liver models have been presented that provide proof-of-concept for the detection of hepatotoxicity, we are only aware of 11 studies that have screened a larger (> 10) number of compounds (**Table 2**). Of these, four models were based on hepatoma cells, four on PHH, and three on hiPS-Heps. Most hepatotoxicity screens are published using spheroids (8/11), whereas only two and one used MPCC and liver chips, respectively. Notably, as of yet, no DILI prediction study with more than 10 compounds was published using bioreactors or bioprinting modalities.

**Table 2:** Overview of published hepatotoxicity screens in organotypic 3D models. Only studies in which ≥10 compounds were analyzed, are listed.

Culture paradigm	Cell system	Number of compounds tested (DILI positive/DILI negative	Toxicity threshold	Exposure time	Results/conclusions	Reference
Scaffold-free spheroid	HepaRG	12 (10/2)	30x c_max_	24 h/7 days single exposure	24 h exposure: 6 out of 10 are predicted correctly for hepatotoxins compared to 5 out of 10 in 2D HepaRG culture; 7 day exposure: 7 out of 10 are predicted correctly for hepatotoxins compared to 6 out of 10 in 2D HepaRG culture.	(Ott et al., 2017)
Scaffold-free spheroid	HepaRG	10 (6/4)	No threshold defined	24 h single exposure/6 days repeated exposure	Repeated exposure for 3D HepaRG spheroid shows higher sensitivity than 24 h single exposure for 3D HepaRG spheroid, 2D HepaRG, and PHH	(Ramaiahgari et al., 2017)
Scaffold-free spheroid	hiPSC-derived HLC	24 (20/4)	No threshold defined	24 h single exposure	HLC spheroid is more sensitive than HepG2 spheroid. However, for acetaminophen and troglitazone, PHH monolayer culture performs better than HLC spheroid	(Takayama et al., 2013)
Scaffold-free spheroid	hiPSC-derived HLC	48 (42/6)	No threshold defined	72 h single exposure	36 out of 42 hepatotoxins elicit detectable toxicity effects. However, 12 anticancer drugs that are generally cytotoxic are also included as DILI positive compounds	(Sirenko et al., 2016)
Scaffold-free spheroid	PHH	110 (69/41)	25x and 100x c_max_	14 days repeated exposure	47.8% sensitivity and 92.7% specificity at 25x C_max_; 59.4% sensitivity and 80.5% specificity at 100x margin of safety	(Proctor et al., 2017)
Scaffold-free spheroid	PHH	123 (70/53)	1x, 5x, and 20x c_max_	14 days repeated exposure	69% sensitivity and 100% specificity at 20x C_max_ exposure concentration using 80% cell viability as threshold	(Vorrink et al., 2018)
Scaffold-based spheroid	HepG2	12 (8/4)	No threshold defined	24 h single exposure/6 days repeated exposure	Repeated exposure for 3D HepG2 spheroid shows higher sensitivity than 24 h single exposure for 3D HepG2 spheroid and 2D HepG2	(Ramaiahgari et al., 2014)
Scaffold-based spheroid	HepaRG	19 (14/5)	100x c_max_	24 h single exposure/7 days repeated exposure	Liver biomatrix scaffold 3D HepaRG spheroid shows higher sensitivity than non-scaffold scaffold 3D HepaRG spheroid; repeated exposure shows higher sensitivity than 24 h single exposure	(Liu et al., 2018)
MPCC	PHH-fibroblast	45 (35/10)	100x c_max_	9 days repeated exposure	PHH-fibroblast: 65.7% sensitivity and 90% specificity. Predictions are based on multiple endpoints	(Khetani et al., 2013)
MPCC	hiPSC-derived HLC-fibroblast	47 (37/10)	100x c_max_	6 days repeated exposure	65% sensitivity and 100% specificity	(Ware et al., 2015)
Perfused liver chip	PHH-fibroblast	19 (12/7)	100x c_max_	24 h single exposure and 13 days repeated exposure	Human mono-culture: 50%. Human co-culture: 83%.	(Novik et al., 2017)

Importantly, whereas the utilized endpoints are homogenous across studies (10/11 studies used ATP quantifications as proxy for cell viability), the analyzed test compounds were highly dissimilar. This is particularly important with regards to the definition of human hepatotoxicity. Most studies focused on compounds with specific hepatotoxic liability, whereas few others used drugs that were generally cytotoxic, such as anthracyclines, taxanes, or colchicine, which complicates model comparisons (Sirenko et al., 2016). Analogously, the use of negative control compounds can differ from non-hepatotoxic structural analogues of hepatotoxic drugs, which can be considered the gold standard for test specificity, to commonly used media additives (e.g., streptomycin or dexamethasone) and dietary components (such as sucrose or sorbitol).

The compound that was most commonly used for the evaluation of hepatotoxicity was acetaminophen. Therapeutic exposure levels of acetaminophen are 139 µM, whereas overdose plasma concentrations higher than 0.7 mM require immediate intervention (Vale and Proudfoot, 1995). Notably, only few liver models could detect acetaminophen toxicity at clinically relevant concentrations (**Table 3**). PHH spheroid cultures were most sensitive and multiple studies have been presented in which acetaminophen toxicity was detected upon repeated exposure for 2 weeks with TC_50_ values <1 mM. Heterogenous results were obtained for HepaRG spheroids. Whereas some studies reported very high sensitivity (TC_50_ around 1 mM; Leite et al., 2016) already after a 24-h exposure, other reports indicated considerably lower sensitivity (7.6 mM; Wang et al., 2015). HepG2 spheroids were considerably less sensitive with TC_50_ values of 7.2 mM after 4 days of repeated exposures. Lastly, two studies in hiPS-Hep spheroids did not detect acetaminophen toxicity within the analyzed concentration range (up to 10–20 mM).

**Table 3:** Comparative overview of the sensitivity of human organotypic liver models to acetaminophen-induced hepatotoxicity.

Cell model	Cell type	Scaffold	Single/repeated exposure	Exposure time	Mono-/co-culture	TC_50_ (mM)	Reference
Spheroid	PHH	No	Repeated	14 days	Monoculture	0.32–0.8	(Bell et al., 2018)
Spheroid	PHH	No	Repeated	14 days	Co-culture with NPCs	0.57	(Proctor et al., 2017)
Spheroid	PHH	No	Repeated	14 days	Monoculture	0.64	(Bell et al., 2017)
Spheroid	PHH	No	Repeated	14 days	Co-culture with NPCs	0.75	(Messner et al., 2013)
Spheroid	HepaRG	No	Single	24 h	Monoculture	∼1.1	(Leite et al., 2016)
Spheroid	PHH	No	Repeated	8 days	Monoculture	1.3	(Hendriks et al., 2016)
Spheroid	HepaRG	No	Repeated	8 days	Monoculture	1.8	(Hendriks et al., 2016)
Spheroid	PHH	No	Repeated	10 days	Co-culture with NPCs	1.7	(Foster et al., 2019)
Perfused liver chip	PHH	Rat tail collagen-I	Repeated	10 days	Co-culture with LSECs	2.4	(Foster et al., 2019)
Spheroid	HepaRG	PEGDA	Single	24 h	Monoculture	2.48	(Wang et al., 2016)
Spheroid	HepaRG	No	Single	24 h	Monoculture	2.7	(Gunness et al., 2013)
Spheroid	HepaRG	No	Repeated	6 days	Monoculture	2.879	(Ramaiahgari et al., 2017)
Spheroid	HepaRG	Liver biomatrix	Repeated	7 days	Monoculture	∼4	(Liu et al., 2018)
MPCC	hiPSC-HLC	Rat tail collagen-I	Repeated	6 days	Co-culture with mouse fibroblasts	4.48	(Ware et al., 2015)
Perfused liver chip	PHH	Rat tail collagen-I	Repeated	13 days	Co-culture with NPCs	5.59	(Novik et al., 2017)
Spheroid	HepG2	No	Repeated	4 days	Monoculture	7.21	(Gaskell et al., 2016)
Spheroid	HepaRG	No	Single	24 h	Monoculture	7.62	(Wang et al., 2015)
Spheroid	HepG2	Hydrogel	Repeated	6 days	Monoculture	∼9.4	(Ramaiahgari et al., 2014)
Spheroid	hiPSC/hESC-HLC	Galactosylated cellulosic sponge	Single	24 h	Monoculture	>10	(Tasnim et al., 2016)
Spheroid	hiPSC-HLC	No	Single	24 h	Monoculture	>20	(Takayama et al., 2013)
Spheroid	HepG2	No	Single	24 h	Monoculture	>20	(Takayama et al., 2013)
MPCC	PHH	Rat tail collagen-I	Single	24 h	Co-culture with mouse fibroblasts	35	(Khetani and Bhatia, 2008)
Spheroid	HepG2	No	Single	24 h	Monoculture	40	(Fey and Wrzesinski, 2012)

Studies are sorted by descending sensitivity. HLC, hepatocyte-like cell; LSEC, liver sinusoidal endothelial cells; NPC, non-parenchymal cell; PHH, primary human hepatocytes; PEGDA, poly-ethylene-glycol-diacrylate.

Interestingly, hiPS-Hep cultured as MPCC were able to detect acetaminophen toxicity albeit at relatively high concentrations (TC_50_ = 4.5 mM), indicating that this culture paradigm might be specifically useful for the cultivation of stem cell-derived cells. However, acute exposure of PHH-MPCC did not result in relevant toxicity (TC_50_ = 35 mM). Liver-on-a-chip studies using acetaminophen as a benchmark have only recently been published and exhibit modest sensitivity (TC_50_ = 2–5.59 mM). Importantly, model sensitivity to acetaminophen increased overall with increasing exposure time up to 2 weeks, whereas acetaminophen hepatotoxicity is clinically most relevant in the context of acute acetaminophen poisoning, which occurs within hours. Acetaminophen depletes the cellular reduced glutathione (GSH) stores and excessive necrosis only manifests once GSH levels drop below 40% (Mitchell et al., 1973). The lack of acetaminophen toxicity at clinical overdose concentrations in the acute setting could be at least in part due to high levels of bioactive supplements in common culture media with antioxidant properties, such as ascorbic acid (vitamin C), retinol (vitamin A), and α-tocopherol (vitamin E), which might delay hepatocellular GSH depletion.

In conclusion, PHH spheroids appear to be the most sensitive model system for the detection of acetaminophen toxicity, followed by liver-on-chip systems. MPCC seem to be particularly useful for the functional support of hiPS-Heps, whereas the sensitivity of PHH-MPCC to acute single dose exposures to acetaminophen is surprisingly low.

## Applications Beyond Hepatotoxicity Testing

### Guidance for the Selection of Animal Safety Models

The safety of drugs and drug candidates can vary markedly between species and toxicity of the hepatobiliary system constitutes one of the areas with most pronounced species differences (Olson et al., 2000). Hepatotoxicity of acetaminophen constitutes arguably the most studied example. Although the metabolic fate of acetaminophen is similar between species, hepatotoxicity manifests in mice and humans whereas rats and cynomolgus monkeys are protected (McGill et al., 2012; Yu et al., 2015). While safety tests in at least one rodent and one non-rodent species are strongly recommended by regulators before a compound can progress into clinical stages, the choice of appropriate animal model is less controlled and often based on historical in-house data or anecdotal evidence.

Importantly, species-specific differences can be mimicked in 3D primary hepatocyte cultures. A recent study demonstrated that primary hepatocytes from commonly used preclinical rodent (mouse and rat) and non-rodent (minipig and rhesus monkey) animal models cultured as spheroids can recapitulate species-specific toxicity at clinically relevant concentrations (Vorrink et al., 2018). The system recapitulated acetaminophen hepatotoxicity in mouse and human hepatocyte spheroids, whereas hepatocytes from rats and primates in identical conditions were protected. These data show that cross-species comparisons of 3D primary hepatocyte spheroids can provide guidance for the selection of the preclinical model species that most closely recapitulates human liver toxicity for the specific compound in question. In contrast, only minor species differences were observed in the HµREL liver chip and acetaminophen toxicity was flagged as hepatotoxic in both human and rat hepatocytes (Novik et al., 2017).

### Metabolite Identification

Hepatic biotransformation of drugs can generate circulating metabolites, which may possess a safety profile different from the parent molecule. Importantly, the panorama of generated metabolites can differ both quantitatively and qualitatively across species. Thus, the identification and safety assessment of metabolites that are found at disproportionate levels in humans compared to animals constitute an important facet of preclinical toxicology. Current regulatory guidance defines disproportionate metabolites that mandate safety evaluation as metabolites that constitute >10% of circulating drug levels in humans and that are either not detected in animals or for which animal exposure is at least 50% lower [ICH M3 (R2), 2010; The US FDA, 2016]. However, certain metabolite classes, such as stable glutathione or N-acetylcysteine conjugates, might be exempt on a case-by-case basis (Luffer-Atlas and Atrakchi, 2017).

3D culture models in which human hepatic cells maintain physiological levels of drug metabolizing enzymes for days to weeks hold promise to improve metabolite profiling compared to conventional systems, such as liver microsomes, liver S9 fractions, hepatocyte suspension cultures, and liver slices (Anderson et al., 2009; Dalvie et al., 2009). Scaffold-free spheroids have been successfully employed to identify phase I and phase II metabolites of diclofenac, midazolam, acetaminophen, and propranolol (Ohkura et al., 2014). Furthermore, the authors could show that their model detected the human specific metabolites lamotrigine-*N*-glucuronide and salbutamol-4-*O*-sulfate, which have not been detected in previous systems. Similarly, PHH MPCC identified 77% (43/56) of the human relevant metabolites of 27 drugs after 7 days of incubation, whereas hepatocyte suspension cultures (55%), S9 fractions (46%), and liver microsomes (39%) detected considerably less (Wang et al., 2010).

Metabolic profiles obtained using hollow fiber bioreactors have been demonstrated to accurately mimic the metabolic fate of compounds *in vivo*. An impressive example is the metabolism of AZD6610, which was primarily hydroxylated in PHH bioreactor cultures and *in vivo*, whereas glucuronidation was major pathway in HepaRG cells in the same bioreactor setup (Darnell et al., 2012). In the same study, the authors identified all major human relevant diclofenac metabolites in both PHH and HepaRG bioreactors.

Phase I and phase II diclofenac metabolites were also identified in the CN Bio LiverChip (Sarkar et al., 2017). This platform was furthermore successfully used for metabolic profiling of hydrocortisone (Sarkar et al., 2015). Moreover, Hultman *et al*. evaluated metabolite formation of quinidine, S-warfarin, metoprolol, acetaminophen, lorazepam, and oxaprozin in hepatocyte suspension cultures and the static HµREL liver chip (Hultman et al., 2016). Notably, of the 20 metabolites identified using HµREL, only 9 were identified in suspension cultures of the same donor. Similar results were obtained in two other studies for the metabolic profiles of timolol, meloxicam, linezolid, and XK469 (Burton et al., 2018), as well as for five additional unspecified test compounds (Cassidy and Yi, 2018). Unfortunately, the sets of test compounds rarely overlap between the different culture paradigms and thus the currently available data do not allow a direct methodological cross-comparison.

### Elucidation of Hepatotoxicity Mechanisms

Comprehensive molecular profiling using omics technologies constitutes a powerful tool box for deciphering the molecular events leading to overt hepatotoxicity of a given drug or drug candidate. However, such studies in 2D monolayer cultures are restricted to the investigation of acute toxicity events at high exposure levels (Grinberg et al., 2014; El-Hachem et al., 2016; Ogese et al., 2019). Long-term stable 3D hepatocyte culture paradigms offer the opportunity to comprehensively profile molecular changes upon long-term exposure at clinically relevant subtoxic doses.

Ware and colleagues used PHH in MPCC as the experimental paradigm to evaluate transcriptomic alterations upon exposure to the hepatotoxins troglitazone, nefazodone, ibufenac, and tolcapone or their nontoxic structural analogues rosiglitazone, buspirone, ibuprofen, and entacapone (Ware et al., 2017). Interestingly, transcriptomic changes in cells treated with hepatotoxins were consistently higher than for the nontoxic analogues, and multiple transcripts were identified that were unique to the liver toxic compounds. Specifically, the authors identified bile acid biosynthesis, fatty acid metabolism, and PPAR signaling as pathways modulated by troglitazone, which correspond to suspected mechanisms of troglitazone-induced hepatotoxicity *in vivo* (Smith, 2003).

In a parallel study, Bell et al. (2017) analyzed the transcriptomic alterations elucidated by three hepatotoxic compounds with different toxicity mechanisms (chlorpromazine, amiodarone, and aflatoxin B1). Importantly, the authors identified both unique and shared toxicity signatures for the compounds that accurately captured the molecular changes observed in patients. The cholestatic agent chlorpromazine significantly downregulated bile acid biosynthesis mirroring inhibition of *CYP7A1* observed in patients with cholestasis, while amiodarone increased PPAR activity, aligning with perturbed fatty acid metabolism *in vivo* (Lauschke and Ingelman-Sundberg, 2016). In contrast, aflatoxin B1 induced genes associated with nucleotide excision repair and DNA replication, matching aflatoxin genotoxicity. In summary, these data indicate that organotypic liver models constitute promising tools for unravelling the molecular underpinnings of mechanistically diverse hepatotoxic liabilities.

## Conclusions and Future Perspectives

Since the publication of the first seminal studies regarding preclinical hepatotoxicity tests using *in vitro* systems, a plethora of different models have been presented that differ in the used culture paradigm, cell model, medium composition, and substratum. Particularly, the development of organotypic and microphysiological liver models using primary liver cells has drastically improved the predictive power of hepatotoxicity tests and a variety of spin-off companies have been founded that aim to facilitate their commercial dissemination (**Table 4**). Furthermore, the possibility to use cellular material from donors with genotypes, phenotypes, or pathologies of interest combined with the possibility to stably maintain these cells for multiple weeks has opened up a multitude of applications beyond the testing of acute liver toxicity (Ingelman-Sundberg and Lauschke, 2018). As outlined in this review, this progress has been made possible by advancements in the field of cell biology, microengineering, and materials science and, as the fields continue to develop, we expect further breakthroughs. Specifically, we anticipate that further optimization of emerging technologies, such as 3D bioprinting, will refine the use of hepatic models for various aspects of liver physiology. Furthermore, the integration of organotypic liver systems with other tissue models in perfusion devices constitutes an important avenue towards *in vitro* systems toxicology (Ewart et al., 2018; Prantil-Baun et al., 2018; Ronaldson-Bouchard and Vunjak-Novakovic, 2018; Wang et al., 2018).

**Table 4:** Overview of companies focusing on organotypic and microphysiological liver models.

Company	Type	Key references describing the model	Key references for hepatotoxicity applications
CN Bio	Liver chip	(Domansky et al., 2010; Vivares et al., 2014)	(Rowe et al., 2018)
Emulate	Liver chip	(Peel et al., 2019)	(Foster et al., 2019)
HepaPredict	Spheroids	(Bell et al., 2016; Vorrink et al., 2017)	(Bell et al., 2017; Vorrink et al., 2018)
Hepregen (now part of BioIVT)	MPCC	(Khetani and Bhatia, 2008; Nguyen et al., 2015)	(Khetani et al., 2013; Ware et al., 2015; Ware et al., 2017)
Hµrel	Liver chip	(Chao et al., 2009; Hegde et al., 2014)	(Novik et al., 2017)
InSphero	Spheroids	(Messner et al., 2013; Messner et al., 2018)	(Proctor et al., 2017)
Organovo	Bioprinting	(Nguyen et al., 2016; Norona et al., 2019)	

Companies are shown in alphabetical order. Note that only references pertaining to primary human liver models are shown.

Notably, while the field of organotypic liver models is developing very rapidly, the adoption of these models into drug development pipelines and regulatory advice lags behind, primarily because systematic validation and benchmarking of most of these novel systems are currently lacking. Comprehensive evaluation of molecular phenotypes and direct comparison to human livers constitutes important information for judging the phenotypic relevance of a model system. Most importantly, there is a need for data that allow drug developers and regulators to directly compare the predictive power of the respective model to the current state of the art and other emerging model systems. To this end, benchmarking of model sensitivity and specificity using a sufficiently large standardized set of prototypic hepatotoxic compounds with diverse toxicity mechanisms would provide important arguments for progressing from proof-of-concept studies towards regulatory acceptance (Roth and Singer, 2014; Lauschke et al., 2016).

## Author Contributions

All authors listed have made a substantial, direct, and intellectual contribution to the work, and approved it for publication.

## Funding

VML is co-founder and owner of HepaPredict AB. The work in the authors’ laboratory is supported by the Swedish Research Council (grant agreement numbers: 2016-01153 and 2016-01154), by the Strategic Research Programme in Diabetes at Karolinska Institutet, by the Lennart Philipson Foundation, and by the Harald and Greta Jeansson Foundation. No writing assistance was utilized in the production of this manuscript.

## Conflict of Interest Statement

VML is co-founder and owner of HepaPredict AB.

The remaining authors declare that the research was conducted in the absence of any commercial or financial relationships that could be construed as a potential conflict of interest.

## References

[B1] Abu-Absi S. F. Friend J. R. Hansen L. K. Hu W.-S. (2002). Structural polarity and functional bile canaliculi in rat hepatocyte spheroids. Exp. Cell Res. 274, 56–67. 10.1006/excr.2001.5467 11855857

[B2] Anderson S. Luffer-Atlas D. Knadler M. P. (2009). Predicting circulating human metabolites: how good are we? Chem. Res. Toxicol. 22, 243–256. 10.1021/tx8004086 19138063

[B3] Axpe E. Oyen M. L. (2016). Applications of alginate-based bioinks in 3D bioprinting. Int. J. Mol. Sci. 17, 1–11. 10.3390/ijms17121976 PMC5187776 27898010

[B4] Baxter M. Withey S. Harrison S. Segeritz C.-P. Zhang F. Atkinson-Dell R. (2015). Phenotypic and functional analyses show stem cell-derived hepatocyte-like cells better mimic fetal rather than adult hepatocytes. J. Hepatol. 62, 581–589. 10.1016/j.jhep.2014.10.016 25457200 PMC4334496

[B5] Beckwitt C. H. Clark A. M. Wheeler S. Taylor D. L. Stolz D. B. Griffith L. (2018). Liver ‘organ on a chip’. Exp. Cell Res. 363, 15–25. 10.1016/j.yexcr.2017.12.023 29291400 PMC5944300

[B6] Bell C. C. Hendriks D. F. G. Moro S. M. L. Ellis E. Walsh J. Renblom A. (2016). Characterization of primary human hepatocyte spheroids as a model system for drug-induced liver injury, liver function and disease. Sci. Rep. 6, 25187. 10.1038/srep25187 27143246 PMC4855186

[B7] Bell C. C. Lauschke V. M. Vorrink S. U. Palmgren H. Duffin R. Andersson T. B. (2017). Transcriptional, Functional, and mechanistic comparisons of stem cell-derived hepatocytes, HepaRG cells, and three-dimensional human hepatocyte spheroids as predictive *in vitro* systems for drug-induced liver injury. Drug Metab. Disposition 45, 419–429. 10.1124/dmd.116.074369 PMC5363699 28137721

[B8] Bell C. C. Dankers A. C. A. Lauschke V. M. Sison-Young R. Jenkins R. Rowe C. (2018). Comparison of hepatic 2D sandwich cultures and 3D spheroids for long-term toxicity applications: a multicenter study. Toxicol. Sci. 162, 655–666. 10.1093/toxsci/kfx289 29329425 PMC5888952

[B9] Benza R. L. Barst R. J. Galie N. Frost A. Girgis R. E. Highland K. B. (2008). Sitaxsentan for the treatment of pulmonary arterial hypertension: a 1-year, prospective, open-label observation of outcome and survival. Chest 134, 775–782. 10.1378/chest.07-0767 18625676

[B10] Berger D. R. Ware B. R. Davidson M. D. Allsup S. R. Khetani S. R. (2015). Enhancing the functional maturity of induced pluripotent stem cell-derived human hepatocytes by controlled presentation of cell–cell interactions *in vitro* . Hepatology 61, 1370–1381. 10.1002/hep.27621 25421237

[B11] Berger B. Donzelli M. Maseneni S. Boess F. Roth A. Krähenbühl S. (2016). Comparison of Liver Cell Models Using the Basel Phenotyping Cocktail. Front. Pharmacol. 7, 443. 10.3389/fphar.2016.00443 27917125 PMC5116554

[B12] Bhise N. S. Manoharan V. Massa S. Tamayol A. Ghaderi M. Miscuglio M. (2016). A liver-on-a-chip platform with bioprinted hepatic spheroids. Biofabrication 8, 014101. 10.1088/1758-5090/8/1/014101 26756674

[B13] Bi Y.-A. Kazolias D. Duignan D. B. (2006). Use of cryopreserved human hepatocytes in sandwich culture to measure hepatobiliary transport. Drug Metab. Dispos. 34, 1658–1665. 10.1124/dmd.105.009118 16782767

[B14] Bierwolf J. Lutgehetmann M. Feng K. Erbes J. Deichmann S. Toronyi E. (2011). Primary rat hepatocyte culture on 3D nanofibrous polymer scaffolds for toxicology and pharmaceutical research. Biotechnol. Bioeng. 108, 141–150. 10.1002/bit.22924 20824672

[B15] Bjornsson E. S. Bergmann O. M. Björnsson H. K. Kvaran R. B. Olafsson S. (2013). Incidence, presentation, and outcomes in patients with drug-induced liver injury in the general population of Iceland. Gastroenterology 144, 1419–1425. 10.1053/j.gastro.2013.02.006 23419359

[B16] Bondesson I. Ekwall B. Hellberg S. Romert L. Stenberg K. Walum E. (1989). MEIC—a new international multicenter project to evaluate the relevance to human toxicity of *in vitro* cytotoxicity tests. Cell Biol. Toxicol. 5, 331–347. 10.1007/BF01795360 2688844

[B17] Brophy C. M. Luebke-Wheeler J. L. Amiot B. P. Khan H. Remmel R. P. Rinaldo P. (2009). Rat hepatocyte spheroids formed by rocked technique maintain differentiated hepatocyte gene expression and function. Hepatology 49, 578–586. 10.1002/hep.22674 19085959 PMC2680349

[B18] Burton R. D. Hieronymus T. Chamem T. Heim D. Anderson S. Zhu X. (2018). Assessment of the biotransformation of low-turnover drugs in the HµREL human hepatocyte coculture model. Drug Metab. Disposition 46, 1617–1625. 10.1124/dmd.118.082867 30135244

[B19] Cassidy K. C. Yi P. (2018). Qualitative and quantitative prediction of human *in vivo* metabolic pathways in a human hepatocyte-murine stromal cell co-culture model. Xenobiotica 48, 1192–1205. 10.1080/00498254.2017.1395927 29143555

[B20] Chan H. F. Zhang Y. Leong K. W. (2016). Efficient One-step production of microencapsulated hepatocyte spheroids with enhanced functions. Small 12, 2720–2730. 10.1002/smll.201502932 27038291 PMC4982767

[B21] Chang T. T. Hughes-Fulford M. (2009). Monolayer and spheroid culture of human liver hepatocellular carcinoma cell line cells demonstrate distinct global gene expression patterns and functional phenotypes. Tiss. Eng. Part A 15, 559–567. 10.1089/ten.tea.2007.0434 PMC6468949 18724832

[B22] Chang R. Nam J. Sun W. (2008). Direct cell writing of 3D microorgan for *in vitro* pharmacokinetic model. Tiss. Eng. Part C: Methods 14, 157–166. 10.1089/ten.tec.2007.0392 18544030

[B23] Chao P. Maguire T. Novik E. Cheng K. C. Yarmush M. L. (2009). Evaluation of a microfluidic based cell culture platform with primary human hepatocytes for the prediction of hepatic clearance in human. Biochem. Pharmacol. 78, 625–632. 10.1016/j.bcp.2009.05.013 19463793 PMC4487512

[B24] Chrisp P. Goa K. L. (1990). Dilevalol. Drugs 39, 234–263. 10.2165/00003495-199039020-00007 2184002

[B25] Clark A. M. Wheeler S. E. Young C. L. Stockdale L. Shepard Neiman J. Zhao W. (2017). A liver microphysiological system of tumor cell dormancy and inflammatory responsiveness is affected by scaffold properties. Lab Chip 17, 156–168. 10.1039/C6LC01171C PMC5242229 27910972

[B26] Cook D. Brown D. Alexander R. March R. Morgan P. Satterthwaite G. (2014). Lessons learned from the fate of AstraZeneca’s drug pipeline: a five-dimensional framework. Nat. Rev. Drug Discov. 13, 419–431. 10.1038/nrd4309 24833294

[B27] Court M. H. (2013). Feline drug metabolism and disposition: pharmacokinetic evidence for species differences and molecular mechanisms. Vet. Clin. North Am. Small Anim. Pract. 43, 1039–1054. 10.1016/j.cvsm.2013.05.002 23890237 PMC3811070

[B28] Cui P. Wang S. (2019). Application of microfluidic chip technology in pharmaceutical analysis—a review. J. Pharmaceut. Anal. 9, 238–247. 10.1016/j.jpha.2018.12.001 PMC6704040 31452961

[B29] Dalvie D. Obach R. S. Kang P. Prakash C. Loi C.-M. Hurst S. (2009). Assessment of three human *in vitro* systems in the generation of major human excretory and circulating metabolites. Chem. Res. Toxicol. 22, 357–368. 10.1021/tx8004357 19146377

[B30] Darnell M. Ulvestad M. Ellis E. Weidolf L. Andersson T. B. (2012). *In vitro* evaluation of major *in vivo* drug metabolic pathways using primary human hepatocytes and HepaRG cells in suspension and a dynamic three-dimensional bioreactor system. J. Pharmacol. Exp. Therapeut. 343, 134–144. 10.1124/jpet.112.195834 22776955

[B31] De Bartolo L. Salerno S. Curcio E. Piscioneri A. Rende M. Morelli S. (2009). Human hepatocyte functions in a crossed hollow fiber membrane bioreactor. Biomaterials 30, 2531–2543. 10.1016/j.biomaterials.2009.01.011 19185912

[B32] De Bruyn T. Chatterjee S. Fattah S. Keemink J. Nicolaï J. Augustijns P. (2013). Sandwich-cultured hepatocytes: utility for *in vitro* exploration of hepatobiliary drug disposition and drug-induced hepatotoxicity. Expert Opin.Drug Metab. Toxicol. 9, 589–616. 10.1517/17425255.2013.773973 23452081

[B33] de Morais S. M. Uetrecht J. P. Wells P. G. (1992). Decreased glucuronidation and increased bioactivation of acetaminophen in Gilbert’s syndrome. Gastroenterology 102, 577–586. 10.1016/0016-5085(92)90106-9 1732127

[B34] Domansky K. Inman W. Serdy J. Dash A. Lim M. H. M. Griffith L. G. (2010). Perfused multiwell plate for 3D liver tissue engineering. Lab Chip 10, 51–58. 10.1039/B913221J 20024050 PMC3972823

[B35] Donato M. T. Martínez-Romero A. Jiménez N. Negro A. Herrera G. Castell J. V. (2009). Cytometric analysis for drug-induced steatosis in HepG2 cells. Chem.-Biol. Interact. 181, 417–423. 10.1016/j.cbi.2009.07.019 19647728

[B36] Ebrahimkhani M. R. Neiman J. A. S. Raredon M. S. B. Hughes D. J. Griffith L. G. (2014) Bioreactor technologies to support liver function *in vitro*. Adv. Drug Deliv. Rev. 69 (70), 132–157. 10.1016/j.addr.2014.02.011 24607703 PMC4144187

[B37] Ekwall B. Barile F. A. Castano A. Clemedson C. Clothier R. H. Dierickx P. (1998). MEIC evaluation of acute systemic toxicity—Part VI. The prediction of human toxicity by rodent LD50 values and results from 61 *in vitro* methods. Altern. Lab. Anim. 26, 617–658. 26042663

[B38] El-Hachem N. Grossmann P. Blanchet-Cohen A. Bateman A. R. Bouchard N. Archambault J. (2016). Characterization of conserved toxicogenomic responses in chemically exposed hepatocytes across species and platforms. Envi. Health Pers. 124, 313–320. 10.1289/ehp.1409157 PMC4786983 26173225

[B39] Elkayam T. Amitay-Shaprut S. Dvir-Ginzberg M. Harel T. Cohen S. (2006). Enhancing the drug metabolism activities of C3A—a human hepatocyte cell line—by tissue engineering within alginate scaffolds. Tiss. Eng. 12, 1357–1368. 10.1089/ten.2006.12.1357 16771648

[B40] Ewart L. Dehne E.-M. Fabre K. Gibbs S. Hickman J. Hornberg E. (2018). Application of microphysiological systems to enhance safety assessment in drug discovery. Annu. Rev. Pharmacol. Toxicol. 580, 65–82. 10.1146/annurev-pharmtox-010617-052722 29029591

[B41] Faulkner-Jones A. Fyfe C. Cornelissen D.-J. Gardner J. King J. Courtney A. (2015). Bioprinting of human pluripotent stem cells and their directed differentiation into hepatocyte-like cells for the generation of mini-livers in 3D. Biofabrication 7, 044102. 10.1088/1758-5090/7/4/044102 26486521

[B42] Fey S. J. Wrzesinski K. (2012). Determination of drug toxicity using 3D spheroids constructed from an immortal human hepatocyte cell line. Toxicol. Sci. 127, 403–411. 10.1093/toxsci/kfs122 22454432 PMC3355318

[B43] Fontana R. J. Hayashi P. H. Gu J. Reddy K. R. Barnhart H. Watkins P. B. (2014). Idiosyncratic drug-induced liver injury is associated with substantial morbidity and mortality within 6 months from onset. Gastroenterology 147, 96–108. 10.1053/j.gastro.2014.03.045 24681128 PMC4285559

[B44] Foppiano M. Lombardo G. (1997). Worldwide pharmacovigilance systems and tolrestat withdrawal. Lancet 349, 399–400. 10.1016/S0140-6736(97)80018-9 9033472

[B45] Foster A. J. Chouhan B. Regan S. L. Rollison H. Amberntsson S. Andersson L. C. (2019). Integrated *in vitro* models for hepatic safety and metabolism: evaluation of a human liver-chip and liver spheroid. Arch. Toxicol. 239, 1180. 10.1007/s00204-019-02427-4 30915487

[B46] Francis C. W. Berkowitz S. D. Comp P. C. Lieberman J. R. Ginsberg J. S. Paiement G. (2003). for the EXULT A Study Group, Comparison of ximelagatran with warfarin for the prevention of venous thromboembolism after total knee replacement. N. Eng. J. Med. 349, 1703–1712. 10.1056/NEJMoa035162 14585938

[B47] Gale E. A. M. (2006). Troglitazone: the lesson that nobody learned? Diabetologia 49, 1–6. 10.1007/s00125-005-0074-6 16362281

[B48] Gaskell H. Sharma P. Colley H. E. Murdoch C. Williams D. P. Webb S. D. (2016). Characterization of a functional C3A liver spheroid model. Toxicol. Res. 5, 1053–1065. 10.1039/C6TX00101G PMC5047049 27746894

[B49] Gerlach J. C. Encke J. Hole O. Muller C. Ryan C. J. Neuhaus P. (1994). Bioreactor for a larger scale hepatocyte *in-vitro* perfusion. Transplantation 58, 984–988. 10.1097/00007890-199411150-00002 7974737

[B50] Gerlach J.r.C. Mutig K. Sauer I. M. Schrade P. Efimova E. Mieder T. (2003). Use of primary human liver cells originating from discarded grafts in a bioreactor for liver support therapy and the prospects of culturing adult liver stem cells in bioreactors: a morphologic study. Transplantation 76, 781–786. 10.1097/01.TP.0000083319.36931.32 14501853

[B51] Goldkind L. Laine L. (2006). A systematic review of NSAIDs withdrawn from the market due to hepatotoxicity: lessons learned from the bromfenac experience. Pharmacoepidemiol. Drug Safety 15, 213–220. 10.1002/pds.1207 16456879

[B52] Goldring C. Antoine D. J. Bonner F. Crozier J. Denning C. Fontana R. J. (2017). Stem cell-derived models to improve mechanistic understanding and prediction of human drug-induced liver injury. Hepatology 65, 710–721. 10.1002/hep.28886 27775817 PMC5266558

[B53] Gómez-Lechón M. J. Tolosa L. Conde I. Donato M. T. (2014). Competency of different cell models to predict human hepatotoxic drugs. Expert Opin.Drug Metab. Toxicol. 10, 1553–1568. 10.1517/17425255.2014.967680 25297626

[B54] Gomèz-Sjoberg R. Leyrat A. A. Houseman B. T. Shokat K. Quake S. R. (2010). Biocompatibility and reduced drug absorption of sol-gel-treated poly(dimethyl siloxane) for microfluidic cell culture applications. Anal. Chem. 82, 8954–8960. 10.1021/ac101870s 20936785 PMC3032040

[B55] Gopinathan J. Noh I. (2018). Recent trends in bioinks for 3D printing. Biomater. Res. 22, 11. 10.1186/s40824-018-0122-1 29636985 PMC5889544

[B56] Graham D. J. Drinkard C. R. Shatin D. Tsong Y. Burgess M. J. (2001). Liver enzyme monitoring in patients treated with troglitazone. JAMA 286, 831–833. 10.1001/jama.286.7.831 11497537

[B57] Grinberg M. Stöber R. M. Edlund K. Rempel E. Godoy P. Reif R. (2014). Toxicogenomics directory of chemically exposed human hepatocytes. Arch. Toxicol. 88, 2261–2287. 10.1007/s00204-014-1400-x 25399406

[B58] Grix T. Ruppelt A. Thomas A. Amler A.-K. Noichl B. P. Lauster R. (2018). Bioprinting perfusion-enabled liver equivalents for advanced organ-on-a-chip applications. Genes 9, 1–15. 10.3390/genes9040176 PMC5924518 29565814

[B59] Gudapati H. Dey M. Ozbolat I. (2016). A comprehensive review on droplet-based bioprinting: past, present and future. Biomaterials 102, 20–42. 10.1016/j.biomaterials.2016.06.012 27318933

[B60] Gungor-Ozkerim P. S. Inci I. Zhang Y. S. Khademhosseini A. Dokmeci M. R. (2018). Bioinks for 3D bioprinting: an overview. Biomater. Sci. 6, 915–946. 10.1039/C7BM00765E 29492503 PMC6439477

[B61] Gunness P. Mueller D. Shevchenko V. Heinzle E. Ingelman-Sundberg M. Noor F. (2013). 3D organotypic cultures of human HepaRG cells: a tool for *in vitro* toxicity studies. Toxicol. Sci. 133, 67–78. 10.1093/toxsci/kft021 23377618

[B62] Hallifax D. Galetin A. Houston J. B. (2008). Prediction of metabolic clearance using fresh human hepatocytes: comparison with cryopreserved hepatocytes and hepatic microsomes for five benzodiazepines. Xenobiotica 38, 353–367. 10.1080/00498250701834665 18340561

[B63] Hart S. N. Li Y. Nakamoto K. Subileau E.-A. Steen D. Zhong X.-B. (2010). A comparison of whole genome gene expression profiles of HepaRG cells and HepG2 cells to primary human hepatocytes and human liver tissues. Drug Metab. Disposition 38, 988–994. 10.1124/dmd.109.031831 PMC2879958 20228232

[B64] Hegde M. Jindal R. Bhushan A. Bale S. S. McCarty W. J. Golberg I. (2014). Dynamic interplay of flow and collagen stabilizes primary hepatocytes culture in a microfluidic platform. Lab Chip 14, 2033–2039. 10.1039/C4LC00071D 24770663 PMC4036071

[B65] Hendriks D. F. G. Fredriksson Puigvert L. Messner S. Mortiz W. Ingelman-Sundberg M. (2016). Hepatic 3D spheroid models for the detection and study of compounds with cholestatic liability. Sci. Rep. 6, 35434. 10.1038/srep35434 27759057 PMC5069690

[B66] Hoffmann S. A. Müller-Vieira U. Biemel K. Knobeloch D. Heydel S. Lübberstedt M. (2012). Analysis of drug metabolism activities in a miniaturized liver cell bioreactor for use in pharmacological studies. Biotechnol. Bioeng. 109, 3172–3181. 10.1002/bit.24573 22688505

[B67] https://www.ich.org/products/guidelines/safety/article/safety-guidelines.html .

[B68] https://www.ema.europa.eu/en/documents/scientific-guideline/reflection-paper-non-clinical-evaluation-drug-induced-liver-injury-dili_en.pdf .

[B69] Hughes B. (2008). Industry concern over EU hepatotoxicity guidance. Nat. Rev. Drug Discov. 7, 719–719. 10.1038/nrd2677 19172685

[B70] Hultman I. Vedin C. Abrahamsson A. Winiwarter S. Darnell M. (2016). Use of HμREL human coculture system for prediction of intrinsic clearance and metabolite formation for slowly metabolized compounds. Mol. Pharmaceut. 13, 2796–2807. 10.1021/acs.molpharmaceut.6b00396 27377099

[B71] Hwang C. M. Sant S. Masaeli M. Kachouie N. N. Zamanian B. Lee S.-H. (2010). Fabrication of three-dimensional porous cell-laden hydrogel for tissue engineering. Biofabrication 2, 035003. 10.1088/1758-5082/2/3/035003 20823504 PMC3282162

[B72] ICH M3 (R2) 2010 Guidance for industry: nonclinical safety studies for the conduct of human clinical trials and marketing authorization for pharmaceuticals. http://www.fda.gov/downloads/drugs/guidances/ucm073246.pdf5 . [Accessed 0804.2019]. 20349552

[B73] Ingelman-Sundberg M. Lauschke V. M. (2018). Human liver spheroids in chemically defined conditions for studies of gene–drug, drug–drug and disease–drug interactions. Pharmacogenomics 19, 1133–1138. 10.2217/pgs-2018-0096 30041575

[B74] Jang M. Kleber A. Ruckelshausen T. Betzholz R. Manz A. (2019). Differentiation of the human liver progenitor cell line (HepaRG) on a microfluidic-based biochip. J. Tiss. Eng. Regen. Med. 13, 482–494. 10.1002/term.2802 30746894

[B75] Jeon H. Kang K. Park S. A. Kim W. D. Paik S. S. Lee S.-H. (2017). Generation of multilayered 3D structures of HepG2 cells using a bio-printing technique. Gut and Liver 11, 121–128. 10.5009/gnl16010 27559001 PMC5221869

[B76] Jin Y. Kim J. Lee J. S. Min S. Kim S. Ahn D.-H. (2018). Vascularized liver organoids generated using induced hepatic tissue and dynamic liver-specific microenvironment as a drug testing platform. Adv. Funct. Mater. 28, 1801954. 10.1002/adfm.201801954

[B77] Kang H.-W. Lee S. J. Ko I. K. Kengla C. Yoo J. J. Atala A. (2016). A 3D bioprinting system to produce human-scale tissue constructs with structural integrity. Nat. Biotechnol. 34, 312–319. 10.1038/nbt.3413 26878319

[B78] Kang K. Kim Y. Jeon H. Lee S. B. Kim J. S. Park S. A. (2018). Three-dimensional bioprinting of hepatic structures with directly converted hepatocyte-like cells. Tiss. Eng. Part A 24, 576–583. 10.1089/ten.tea.2017.0161 28726547

[B79] Kappos L. Wiendl H. Selmaj K. Arnold D. L. Havrdova E. Boyko A. (2015). Daclizumab HYP versus interferon beta-1a in relapsing multiple sclerosis. N. Eng. J. Med. 373, 1418–1428. 10.1056/NEJMoa1501481 26444729

[B80] Khalil M. Shariat-Panahi A. Tootle R. Ryder T. McCloskey P. Roberts E. (2001). Human hepatocyte cell lines proliferating as cohesive spheroid colonies in alginate markedly upregulate both synthetic and detoxificatory liver function. J. Hepatol. 34, 68–77. 10.1016/S0168-8278(00)00080-5 11211910

[B81] Khetani S. R. Bhatia S. N. (2008). Microscale culture of human liver cells for drug development. Nat. Biotechnol. 26, 120–126. 10.1038/nbt1361 18026090

[B82] Khetani S. R. Kanchagar C. Ukairo O. Krzyzewski S. Moore A. Shi J. (2013). Use of micropatterned cocultures to detect compounds that cause drug-induced liver injury in humans. Toxicol. Sci. 132, 107–117. 10.1093/toxsci/kfs326 23152190

[B83] Kietzmann T. (2017). Metabolic zonation of the liver: the oxygen gradient revisited. Redox Biol. 11, 622–630. 10.1016/j.redox.2017.01.012 28126520 PMC5257182

[B84] Kimoto E. Walsky R. Zhang H. Bi Y. A. Whalen K. M. Yang Y. S. (2012). Differential modulation of cytochrome P450 activity and the effect of 1-aminobenzotriazole on hepatic transport in sandwich-cultured human hepatocytes. Drug Metab. Disposition 40, 407–411. 10.1124/dmd.111.039297 22031626

[B85] Kizawa H. Nagao E. Shimamura M. Zhang G. Torii H. (2017). Scaffold-free 3D bio-printed human liver tissue stably maintains metabolic functions useful for drug discovery. Biochem. Biophys. Rep. 10, 186–191. 10.1016/j.bbrep.2017.04.004 28955746 PMC5614670

[B86] Knowlton S. Tasoglu S. (2016). A bioprinted liver-on-a-chip for drug screening applications. Trends Biotechnol. 34, 681–682. 10.1016/j.tibtech.2016.05.014 27291461

[B87] Koide N. Shinji T. Tanabe T. Asano K. Kawaguchi M. Sakaguchi K. (1989). Continued high albumin production by multicellular spheroids of adult rat hepatocytes formed in the presence of liver-derived proteoglycans. Biochem. Biophys. Res. Commun. 161, 385–391. 10.1016/0006-291X(89)91609-4 2730666

[B88] Kozyra M. Johansson I. Nordling Å. Ullah S. Lauschke V. M. Ingelman-Sundberg M. (2018). Human hepatic 3D spheroids as a model for steatosis and insulin resistance. Sci. Rep. 8, 14297. 10.1038/s41598-018-32722-6 30250238 PMC6155201

[B89] Kyffin J. A. Sharma P. Leedale J. Colley H. E. Murdoch C. Harding A. L. (2019). Characterisation of a functional rat hepatocyte spheroid model. Toxicol. in Vitro 55, 160–172. 10.1016/j.tiv.2018.12.014 30578835 PMC6361770

[B90] Landry J. Bernier D. Ouellet C. Goyette R. Marceau N. (1985). Spheroidal aggregate culture of rat liver cells: histotypic reorganization, biomatrix deposition, and maintenance of functional activities. J. Cell Biol. 101, 914–923. 10.1083/jcb.101.3.914 2411740 PMC2113699

[B91] Lauschke V. M. Ingelman-Sundberg M. (2016). The importance of patient-specific factors for hepatic drug response and toxicity. Int. J. Mol. Sci. 17, 1–27. 10.3390/ijms17101714 PMC5085745 27754327

[B92] Lauschke V. M. Hendriks D. F. G. Bell C. C. Andersson T. B. Ingelman-Sundberg M. (2016). Novel 3D culture systems for studies of human liver function and assessments of the hepatotoxicity of drugs and drug candidates. Chem. Res. Toxicol. 29, 1936–1955. 10.1021/acs.chemrestox.6b00150 27661221

[B93] Lauschke V. M. Vorrink S. U. Moro S. M. L. Rezayee F. Nordling Å. Hendriks D. F. G. (2016). Massive rearrangements of cellular MicroRNA signatures are key drivers of hepatocyte dedifferentiation. Hepatology 64, 1743–1756. 10.1002/hep.28780 27532775

[B94] Lauschke V. M. Shafagh R. Z. Hendriks D. F. G. Ingelman-Sundberg M. (2019). 3D primary hepatocyte culture systems for analyses of liver diseases, drug metabolism and toxicity: emerging culture paradigms and applications. Biotechnol. J. 2, e1800347. 10.1002/biot.201800347 30957976

[B95] Lee J. W. Choi Y.-J. Yong W.-J. Pati F. Shim J.-H. Kang K. S. (2016). Development of a 3D cell printed construct considering angiogenesis for liver tissue engineering. Biofabrication 8, 015007. 10.1088/1758-5090/8/1/015007 26756962

[B96] Lee-Montiel F. T. George S. M. Gough A. H. Sharma A. D. Wu J. DeBiasio R. (2017). Control of oxygen tension recapitulates zone-specific functions in human liver microphysiology systems. Exp. Biol. Med. 242, 1617–1632. 10.1177/1535370217703978 PMC5661766 28409533

[B97] Leite S. B. Wilk-Zasadna I. Zaldivar J. M. Airola E. Reis-Fernandes M. A. Mennecozzi M. (2012). Three-dimensional HepaRG model as an attractive tool for toxicity testing. Toxicol. Sci. 130, 106–116. 10.1093/toxsci/kfs232 22843569

[B98] Leite S. B. Roosens T. El Taghdouini A. Mannaerts I. Smout A. J. Najimi M. (2016). Novel human hepatic organoid model enables testing of drug-induced liver fibrosis *in vitro* . Biomaterials 78, 1–10. 10.1016/j.biomaterials.2015.11.026 26618472

[B99] Li N. Schwartz M. Ionescu-Zanetti C. (2009). PDMS compound adsorption in context. J. Biomol. Screening 14, 194–202. 10.1177/1087057108327326 19196703

[B100] Lin C. Khetani S. R. (2016). Advances in engineered liver models for investigating drug-induced liver injury. BioMed Res. Int. 2016, 1829148. 10.1155/2016/1829148 27725933 PMC5048025

[B101] Liu J. Li R. Xue R. Li T. Leng L. Wang Y. (2018). Liver Extracellular matrices bioactivated hepatic spheroids as a model system for drug hepatotoxicity evaluations. Adv. Biosyst. 2, 1800110–14. 10.1002/adbi.201800110

[B102] Long T. J. Cosgrove P. A. Dunn R. T. Stolz D. B. Hamadeh H. Afshari C. (2016). Modeling therapeutic antibody-small molecule drug–drug interactions using a three-dimensional perfusable human liver coculture platform. Drug Metab. Disposition 44, 1940–1948. 10.1124/dmd.116.071456 PMC5118635 27621203

[B103] Lübberstedt M. Müller-Vieira U. Biemel K. M. Darnell M. Hoffmann S. A. Knöspel F. (2015). Serum-free culture of primary human hepatocytes in a miniaturized hollow-fibre membrane bioreactor for pharmacological *in vitro* studies. J. Tiss. Eng. Regen. Med. 9, 1017–1026. 10.1002/term.1652 23165723

[B104] Luckert C. Schulz C. Lehmann N. Thomas M. Hofmann U. Hammad S. (2017) Comparative analysis of 3D culture methods on human HepG2 cells. Archives of Toxicology. 91, 393–406. 10.1007/s00204-016-1677-z 26872951

[B105] Luffer-Atlas D. Atrakchi A. (2017). A decade of drug metabolite safety testing: industry and regulatory shared learning. Expert Opin.Drug Metab. Toxicol. 13, 897–900. 10.1080/17425255.2017.1364362 28797172

[B106] Ma X. Qu X. Zhu W. Li Y.-S. Yuan S. Zhang H. (2016). Deterministically patterned biomimetic human iPSC-derived hepatic model via rapid 3D bioprinting. Proc. Natl. Acad. Sci. 113, 2206–2211. 10.1073/pnas.1524510113 26858399 PMC4776497

[B107] Marcinak J. F. Munsaka M. S. Watkins P. B. Ohira T. Smith N. (2018). Liver safety of fasiglifam (TAK-875) in patients with type 2 diabetes: review of the global clinical trial experience. Drug Safety 41, 625–640. 10.1007/s40264-018-0642-6 29492878

[B108] Martignoni M. Groothuis G. M. M. de Kanter R. (2006). Species differences between mouse, rat, dog, monkey and human CYP-mediated drug metabolism, inhibition and induction. Expert Opin.Drug Metab. Toxicol. 2, 875–894. 10.1517/17425255.2.6.875 17125407

[B109] Matsusaki M. Sakaue K. Kadowaki K. Akashi M. (2013). Three-dimensional human tissue chips fabricated by rapid and automatic inkjet cell printing. Adv. Healthcare Mater. 2, 534–539. 10.1002/adhm.201200299 23184899

[B110] McDonald G. R. Hudson A. L. Dunn S. M. J. You H. Baker G. B. Whittal R. M. (2008). Bioactive contaminants leach from disposable laboratory plasticware. Science 322, 917. 10.1126/science.1162395 18988846

[B111] McGill M. R. Williams C. D. Xie Y. Ramachandran A. Jaeschke H. (2012). Acetaminophen-induced liver injury in rats and mice: comparison of protein adducts, mitochondrial dysfunction, and oxidative stress in the mechanism of toxicity. Toxicol. Appl. Pharmacol. 264, 387–394. 10.1016/j.taap.2012.08.015 22980195 PMC3478469

[B112] McGinnity D. F. Soars M. G. Urbanowicz R. A. Riley R. J. (2004). Evaluation of fresh and cryopreserved hepatocytes as *in vitro* drug metabolism tools for the prediction of metabolic clearance. Drug Metab. Disposition 32, 1247–1253. 10.1124/dmd.104.000026 15286053

[B113] Meier F. Freyer N. Brzeszczynska J. Knöspel F. Armstrong L. Lako M. (2017). Hepatic differentiation of human iPSCs in different 3D models: a comparative study. Int. J. Mol. Med. 40, 1759–1771. 10.3892/ijmm.2017.3190 29039463 PMC5716452

[B114] Messner S. Agarkova I. Moritz W. Kelm J. M. (2013). Multi-cell type human liver microtissues for hepatotoxicity testing. Arch. Toxicol. 87, 209–213. 10.1007/s00204-012-0968-2 23143619 PMC3535351

[B115] Messner S. Fredriksson L. Lauschke V. M. Roessger K. Escher C. Bober M. (2018). Transcriptomic, proteomic, and functional long-term characterization of multicellular three-dimensional human liver microtissues. Appl. In Vitro Toxicol. 4, 1–12. 10.1089/aivt.2017.0022 PMC7500040 32953943

[B116] Mitchell J. R. Jollow D. J. Potter W. Z. Gillette J. R. Brodie B. B. (1973). Acetaminophen-induced hepatic necrosis. J. Pharmacol. Exp. Therapeut. 187, 211–217. 4746329

[B117] Mueller D. Krämer L. Hoffmann E. Klein S. Noor F. (2014). 3D organotypic HepaRG cultures as *in vitro* model for acute and repeated dose toxicity studies. Toxicol. in Vitro 28, 104–112. 10.1016/j.tiv.2013.06.024 23850736

[B118] Mun S. J. Ryu J.-S. Lee M.-O. Son Y. S. Oh S. J. Cho H.-S. (2019). Generation of expandable human pluripotent stem cell-derived hepatocyte-like liver organoids. J. Hepatol. 10.1016/j.jhep.2019.06.030. [Epub ahead of print]. 31299272

[B119] Natarajan V. Berglund E. J. Chen D. X. Kidambi S. (2015). Substrate stiffness regulates primary hepatocyte functions. RSC Advances 5, 80956–80966. 10.1039/C5RA15208A PMC7392243 32733675

[B120] Nelson L. J. Morgan K. Treskes P. Samuel K. Henderson C. J. LeBled C. (2017). Human hepatic HepaRG cells maintain an organotypic phenotype with high intrinsic CYP450 activity/metabolism and significantly outperform standard HepG2/C3A cells for pharmaceutical and therapeutic applications. Basic Clin. Pharmacol. Toxicol. 120, 30–37. 10.1111/bcpt.12631 27285124 PMC5225883

[B121] Nguyen T. V. Ukairo O. Khetani S. R. McVay M. Kanchagar C. Seghezzi W. (2015). Establishment of a hepatocyte-Kupffer cell coculture model for assessment of proinflammatory cytokine effects on metabolizing enzymes and drug transporters. Drug Metab. Disposition 43, 774–785. 10.1124/dmd.114.061317 25739975

[B122] Nguyen D. G. Funk J. Robbins J. B. Crogan-Grundy C. Presnell S. C. Singer T. (2016). Bioprinted 3D primary liver tissues allow assessment of organ-level response to clinical drug induced toxicity *in vitro* . PLoS One 11, e0158674–17. 10.1371/journal.pone.0158674 PMC4936711 27387377

[B123] Nichols W. G. Steel H. M. Bonny T. Adkison K. Curtis L. Millard J. (2008). Hepatotoxicity observed in clinical trials of aplaviroc (GW873140). Antimicrob. Agents Chemother. 52, 858–865. 10.1128/AAC.00821-07 18070967 PMC2258506

[B124] Norona L. M. Nguyen D. G. Gerber D. A. Presnell S. C. Mosedale M. Watkins P. B. (2019). Bioprinted liver provides early insight into the role of Kupffer cells in TGF-β1 and methotrexate-induced fibrogenesis. PLoS One 14, e0208958. 10.1371/journal.pone.0208958 30601836 PMC6314567

[B125] Novik E. Maguire T. J. Chao P. Cheng K. C. Yarmush M. L. (2010). A microfluidic hepatic coculture platform for cell-based drug metabolism studies. Biochem. Pharmacol. 79, 1036–1044. 10.1016/j.bcp.2009.11.010 19925779 PMC3136813

[B126] Novik E. I. Dwyer J. Morelli J. K. Parekh A. Cho C. Pludwinski E. (2017). Long-enduring primary hepatocyte-based co-cultures improve prediction of hepatotoxicity. Toxicol. Appl. Pharmacol. 336, 20–30. 10.1016/j.taap.2017.09.013 28942002

[B127] Ogese M. O. Jenkins R. E. Adair K. Tailor A. Meng X. Faulkner L. L. (2019). Exosomal transport of hepatocyte-derived drug-modified proteins to the immune system. Hepatology. 10.1002/hep.30701. [Epub ahead of print]. PMC6899733 31070244

[B128] Ogihara T. Iwai H. Inoue Y. Katagi J. Matsumoto N. Motoi-Ohtsuji M. (2015). Utility of human hepatocyte spheroids for evaluation of hepatotoxicity. Fundament. Toxicol. Sci. 2, 41–48. 10.2131/fts.2.41

[B129] Ogihara T. Arakawa H. Jomura T. Idota Y. Koyama S. Yano K. (2017). Utility of human hepatocyte spheroids without feeder cells for evaluation of hepatotoxicity. J. Toxicol. Sci. 42, 499–507. 10.2131/jts.42.499 28717109

[B130] Ohkura T. Ohta K. Nagao T. Kusumoto K. Koeda A. Ueda T. (2014). Evaluation of human hepatocytes cultured by three-dimensional spheroid systems for drug metabolism. Drug Metab. Pharmacokinet. 29, 373–378. 10.2133/dmpk.DMPK-13-RG-105 24695277

[B131] Olanow C. W. Panel T. A. (2000). Tolcapone and hepatotoxic effects. Arch. Neurol. 57, 263–267. 10.1001/archneur.57.2.263 10681087

[B132] Olson H. Betton G. Robinson D. Thomas K. Monro A. Kolaja G. (2000). Concordance of the toxicity of pharmaceuticals in humans and in animals. Regul. Toxicol. Pharmacol. 32, 56–67. 10.1006/rtph.2000.1399 11029269

[B133] Oorts M. Baze A. Bachellier P. Heyd B. Zacharias T. Annaert P. (2016). Drug-induced cholestasis risk assessment in sandwich-cultured human hepatocytes. Toxicol. in Vitro 34, 179–186. 10.1016/j.tiv.2016.03.008 27046439

[B134] Ostapowicz G. Fontana R. J. Schiødt F. V. Larson A. Davern T. J. Han S. H. B. (2002). Results of a prospective study of acute liver failure at 17 tertiary care centers in the United States. Ann.Inter. Med. 137, 947–954. 10.7326/0003-4819-137-12-200212170-00007 12484709

[B135] Ott L. M. Ramachandran K. Stehno-Bittel L. (2017). An automated multiplexed hepatotoxicity and CYP induction assay using HepaRG cells in 2D and 3D. SLAS Discovery 22, 614–625. 10.1177/2472555217701058 28346810

[B136] Ozbolat I. T. Hospodiuk M. (2016) Current advances and future perspectives in extrusion-based bioprinting. Biomaterials 76, 321–343 10.1016/j.biomaterials.2015.10.076 26561931

[B137] Peel S. Corrigan A. M. Ehrhardt B. Jang K.-J. Caetano-Pinto P. Boeckeler M. (2019). Introducing an automated high content confocal imaging approach for organs-on-chips. Lab Chip 19, 410–421. 10.1039/C8LC00829A 30663729

[B138] Petersen P. Grind M. Adler J. (2003). for the SPORTIF II Investigators, Ximelagatran versus warfarin for stroke prevention in patients with nonvalvular atrial fibrillation. J. Am. Coll. Cardiol. 41, 1445–1451. 10.1016/S0735-1097(03)00255-9 12742279

[B139] Phillips M. B. Balbuena-Venancio P. Enders J. R. Norini R. L. Shim Y.-S. Burgunder E. (2018). Xenobiotic metabolism in alginate-encapsulated primary human hepatocytes over long timeframes. Appl. In Vitro Toxicol. 4, 238–247. 10.1089/aivt.2017.0029

[B140] Planchamp C. Vu T. L. Mayer J. M. Reist M. Testa B. (2003). Hepatocyte hollow-fibre bioreactors: design, set-up, validation and applications. J. Pharm. Pharmacol. 55, 1181–1198. 10.1211/0022357021963 14604461

[B141] Pless G. Steffen I. Zeilinger K. Sauer I. M. Katenz E. Kehr D. C. (2006). Evaluation of primary human liver cells in bioreactor cultures for extracorporeal liver support on the basis of urea production. Artif. Organs 30, 686–694. 10.1111/j.1525-1594.2006.00285.x 16934097

[B142] Powers M. J. Domansky K. Kaazempur-Mofrad M. R. Kalezi A. Capitano A. Upadhyaya A. (2002). A microfabricated array bioreactor for perfused 3D liver culture. Biotechnol. Bioeng. 78, 257–269. 10.1002/bit.10143 11920442

[B143] Prantil-Baun R. Novak R. Das D. Somayaji M. R. Przekwas A. Ingber D. E. (2018). Physiologically based pharmacokinetic and pharmacodynamic analysis enabled by microfluidically linked organs-on-chips. Annu. Rev. Pharmacol. Toxicol. 58, 37–64. 10.1146/annurev-pharmtox-010716-104748 29309256

[B144] Proctor W. R. Foster A. J. Vogt J. Summers C. Middleton B. Pilling M. A. (2017). Utility of spherical human liver microtissues for prediction of clinical drug-induced liver injury. Arch. Toxicol. 91, 2849–2863. 10.1007/s00204-017-2002-1 28612260 PMC5515971

[B145] Ramaiahgari S. C. Braver M. W. Herpers B. Terpstra V. Commandeur J. N. M. de Water B. (2014). A 3D *in vitro* model of differentiated HepG2 cell spheroids with improved liver-like properties for repeated dose high-throughput toxicity studies. Arch. Toxicol. 88, 1083–1095. 10.1007/s00204-014-1215-9 24599296

[B146] Ramaiahgari S. C. Waidyanatha S. Dixon D. DeVito M. J. Paules R. S. Ferguson S. S. (2017). Three-Dimensional (3D) HepaRG spheroid model with physiologically relevant xenobiotic metabolism competence and hepatocyte functionality for liver toxicity screening. Toxicol. Sci. 159, 124–136. 10.1093/toxsci/kfx122 28633424 PMC5837526

[B147] Rebelo S. P. Costa R. Estrada M. Shevchenko V. Brito C. Alves P. M. (2015). HepaRG microencapsulated spheroids in DMSO-free culture: novel culturing approaches for enhanced xenobiotic and biosynthetic metabolism. Arch. Toxicol. 89, 1347–1358. 10.1007/s00204-014-1320-9 25107451

[B148] Rebelo S. P. Costa R. Silva M. M. Marcelino P. Brito C. Alves P. M. (2017). Three-dimensional co-culture of human hepatocytes and mesenchymal stem cells: improved functionality in long-term bioreactor cultures. J. Tiss. Eng. Regen. Med. 11, 2034–2045. 10.1002/term.2099 26511086

[B149] Richert L. Baze A. Parmentier C. Gerets H. H. J. Sison-Young R. Dorau M. (2016). Cytotoxicity evaluation using cryopreserved primary human hepatocytes in various culture formats. Toxicol. Lett. 258, 207–215. 10.1016/j.toxlet.2016.06.1127 27363785

[B150] Ronaldson-Bouchard K. Vunjak-Novakovic G. (2018). Organs-on-a-chip: a fast track for engineered human tissues in drug development. Cell Stem Cell 22, 310–324. 10.1016/j.stem.2018.02.011 29499151 PMC5837068

[B151] Roth A. Singer T. (2014). The application of 3D cell models to support drug safety assessment: opportunities & challenges. Adv. Drug Delivery Rev. 69 (70), 179–189. 10.1016/j.addr.2013.12.005 24378580

[B152] Rowe C. Gerrard D. T. Jenkins R. Berry A. Durkin K. Sundstrom L. (2013). Proteome-wide analyses of human hepatocytes during differentiation and dedifferentiation. Hepatology 58, 799–809. 10.1002/hep.26414 23526496 PMC3842115

[B153] Rowe C. Shaeri M. Large E. Cornforth T. Robinson A. Kostrzewski T. (2018). Perfused human hepatocyte microtissues identify reactive metabolite-forming and mitochondria-perturbing hepatotoxins. Toxicol. in Vitro 46, 29–38. 10.1016/j.tiv.2017.09.012 28919358

[B154] Safer D. J. Zito J. M. Gardner J. F. (2001). Pemoline hepatotoxicity and postmarketing surveillance. J. Am. Acad. Child Adol. Psychiatry 40, 622–629. 10.1097/00004583-200106000-00006 11392339

[B155] Sanoh S. Santoh M. Takagi M. Kanayama T. Sugihara K. Kotake Y. (2014). Fluorometric assessment of acetaminophen-induced toxicity in rat hepatocyte spheroids seeded on micro-space cell culture plates. Toxicol. in Vitro 28, 1176–1182. 10.1016/j.tiv.2014.05.007 24878114

[B156] Sarkar U. Rivera-Burgos D. Large E. M. Hughes D. J. Ravindra K. C. Dyer R. L. (2015). Metabolite profiling and pharmacokinetic evaluation of hydrocortisone in a perfused three-dimensional human liver bioreactor. Drug Metab. Disposition 43, 1091–1099. 10.1124/dmd.115.063495 PMC4468434 25926431

[B157] Sarkar U. Ravindra K. C. Large E. Young C. L. Rivera-Burgos D. Yu J. (2017). Integrated assessment of diclofenac biotransformation, pharmacokinetics, and omics-based toxicity in a three-dimensional human liver-immunocompetent coculture system. Drug Metab. Disposition 45, 855–866. 10.1124/dmd.116.074005 PMC5469400 28450578

[B158] Sauer I. M. Zeilinger K. Pless G. Kardassis D. Theruvath T. Pascher A. (2003). Extracorporeal liver support based on primary human liver cells and albumin dialysis—treatment of a patient with primary graft non-function. J. Hepatol. 39, 649–653. 10.1016/S0168-8278(03)00348-9 12971979

[B159] Schepers A. Li C. Chhabra A. Seney B. T. Bhatia S. (2016). Engineering a perfusable 3D human liver platform from iPS cells. Lab Chip 16, 2644–2653. 10.1039/C6LC00598E 27296616 PMC5318999

[B160] Schnitzer T. J. Burmester G. R. Mysler E. Hochberg M. C. Doherty M. Ehrsam E. (2004). Comparison of lumiracoxib with naproxen and ibuprofen in the Therapeutic Arthritis Research and Gastrointestinal Event Trial (TARGET), reduction in ulcer complications: randomised controlled trial. Lancet 364, 665–674. 10.1016/S0140-6736(04)16893-1 15325831

[B161] Schutte M. Fox B. Baradez M.-O. Devonshire A. Minguez J. Bokhari M. (2011). Rat Primary hepatocytes show enhanced performance and sensitivity to acetaminophen during three-dimensional culture on a polystyrene scaffold designed for routine use. Assay Drug Dev. Technol. 9, 475–486. 10.1089/adt.2011.0371 21675871

[B162] Schwartz R. E. Fleming H. E. Khetani S. R. Bhatia S. N. (2014). Pluripotent stem cell-derived hepatocyte-like cells. Biotechnol. Adv. 32, 504–513. 10.1016/j.biotechadv.2014.01.003 24440487 PMC4043206

[B163] Selden C. Khalil M. Hodgson H. (2000). Three dimensional culture upregulates extracellular matrix protein expression in human liver cell lines—a step towards mimicking the liver *in vivo*? Int. J. Artif. Organs 23, 774–781. 10.1177/039139880002301107 11132022

[B164] Sgro C. Clinard F. Ouazir K. Chanay H. Allard C. Guilleminet C. (2002). Incidence of drug-induced hepatic injuries: a French population-based study. Hepatology 36, 451–455. 10.1053/jhep.2002.34857 12143055

[B165] Shen C. Zhang G. Qiu H. Meng Q. (2006). Acetaminophen-induced hepatotoxicity of gel entrapped rat hepatocytes in hollow fibers. Chem.-Biol. Interact. 162, 53–61. 10.1016/j.cbi.2006.05.005 16797510

[B166] Shen C. Zhang G. Meng Q. (2010). Enhancement of the predicted drug hepatotoxicity in gel entrapped hepatocytes within polysulfone-g-poly (ethylene glycol) modified hollow fiber. Toxicol. Appl. Pharmacol. 249, 140–147. 10.1016/j.taap.2010.08.028 20816885

[B167] Sirenko O. Hancock M. K. Hesley J. Hong D. Cohen A. Gentry J. (2016). Phenotypic characterization of toxic compound effects on liver spheroids derived from iPSC using confocal imaging and three-dimensional image analysis. Assay Drug Dev. Technol. 14, 381–394. 10.1089/adt.2016.729 27494736 PMC5003004

[B168] Sison-Young R. L. Lauschke V. M. Johann E. Alexandre E. Anthérieu S. Aerts H. (2017). A multicenter assessment of single-cell models aligned to standard measures of cell health for prediction of acute hepatotoxicity. Arch. Toxicol. 91, 1385–1400. 10.1007/s00204-016-1745-4 27344343 PMC5316403

[B169] Skardal A. Devarasetty M. Kang H.-W. Mead I. Bishop C. Shupe T. (2015). A hydrogel bioink toolkit for mimicking native tissue biochemical and mechanical properties in bioprinted tissue constructs. Acta Biomater. 25, 24–34. 10.1016/j.actbio.2015.07.030 26210285

[B170] Smith M. T. (2003). Mechanisms of troglitazone hepatotoxicity. Chem. Res. Toxicol. 16, 679–687. 10.1021/tx034033e 12807350

[B171] Spigset O. Hägg S. Bate A. (2003). Hepatic injury and pancreatitis during treatment with serotonin reuptake inhibitors: data from the World Health Organization (WHO) database of adverse drug reactions. Int. Clin. Psychopharmacol. 18, 157–161. 10.1097/01.yic.0000066455.73432.d2 12702895

[B172] Swift B. Pfeifer N. D. Brouwer K. L. R. (2010). Sandwich-cultured hepatocytes: an *in vitro* model to evaluate hepatobiliary transporter-based drug interactions and hepatotoxicity. Drug Metab. Rev. 42, 446–471. 10.3109/03602530903491881 20109035 PMC3097390

[B173] Takahashi Y. Hori Y. Yamamoto T. Urashima T. Ohara Y. Tanaka H. (2015). Three-dimensional (3D) spheroid cultures improve the metabolic gene expression profiles of HepaRG cells. Biosci. Rep. 35, 1–7. 10.1042/BSR20150034 PMC4613666 26182370

[B174] Takayama K. Kawabata K. Nagamoto Y. Kishimoto K. Tashiro K. Sakurai F. (2013). 3D spheroid culture of hESC/hiPSC-derived hepatocyte-like cells for drug toxicity testing. Biomaterials 34, 1781–1789. 10.1016/j.biomaterials.2012.11.029 23228427

[B175] Tan K. Keegan P. Rogers M. Lu M. Gosset J. R. Charest J. (2019). A high-throughput microfluidic microphysiological system (PREDICT-96) to recapitulate hepatocyte function in dynamic, re-circulating flow conditions. Lab Chip 19, 1556–1566. 10.1039/C8LC01262H 30855604

[B176] Tasnim F. Toh Y.-C. Qu Y. Li H. Phan D. Narmada B. C. (2016). Functionally enhanced human stem cell derived hepatocytes in galactosylated cellulosic sponges for hepatotoxicity testing. Mol. Pharmaceut. 13, 1947–1957. 10.1021/acs.molpharmaceut.6b00119 27157693

[B177] The US FDA (2016). Guidance for industry: safety testing of drug metabolites. http://www.fda.gov/downloads/drugs/guidancecomplianceregulatoryinformation/guidances/ucm079266.pdf . [Accessed 08.04.2019].

[B178] Toepke M. W. Beebe D. J. (2006). PDMS absorption of small molecules and consequences in microfluidic applications. Lab Chip 6, 1484–1486. 10.1039/b612140c 17203151

[B179] Tomlinson L. Hyndman L. Firman J. W. Bentley R. Kyffin J. A. Webb S. D. (2019). *In vitro* liver zonation of primary rat hepatocytes. Front. Bioeng. Biotechnol. 7, 253–258. 10.3389/fbioe.2019.00017 PMC6387900 30834246

[B180] Tong J. Z. De Lagausie P. Furlan V. Cresteil T. Bernard O. Alvarez F. (1992). Long-term culture of adult rat hepatocyte spheroids. Exp. Cell Res. 200, 326–332. 10.1016/0014-4827(92)90179-C 1572400

[B181] Tostões R. M. Leite S. B. Serra M. Jensen J. Björquist P. Carrondo M. J. T. (2012). Human liver cell spheroids in extended perfusion bioreactor culture for repeated-dose drug testing. Hepatology 55, 1227–1236. 10.1002/hep.24760 22031499

[B182] Trepanier L. A. Ray K. Winand N. J. Spielberg S. P. Cribb A. E. (1997). Cytosolic arylamine N-acetyltransferase (NAT) deficiency in the dog and other canids due to an absence of NAT genes. Biochem. Pharmacol. 54, 73–80. 10.1016/S0006-2952(97)00140-8 9296352

[B183] Ulvestad M. Darnell M. Molden E. Ellis E. Åsberg A. Andersson T. B. (2012). Evaluation of organic anion-transporting polypeptide 1B1 and CYP3A4 activities in primary human hepatocytes and HepaRG cells cultured in a dynamic three-dimensional bioreactor system. J. Pharmacol. Exp. Therapeut. 343, 145–156. 10.1124/jpet.112.195750 22789711

[B184] Underhill G. H. Khetani S. R. (2018). Bioengineered liver models for drug testing and cell differentiation studies. Cell Mol. Gastroenterol. Hepatol 5, 426–439. 10.1016/j.jcmgh.2017.11.012 29675458 PMC5904032

[B185] Vale J. A. Proudfoot A. T. (1995). Paracetamol (acetaminophen) poisoning. Lancet 346, 547–552. 10.1016/S0140-6736(95)91385-8 7658783

[B186] Vivares A. Salle-Lefort S. Arabeyre-Fabre C. Ngo R. Penarier G. Bremond M. (2014). Morphological behaviour and metabolic capacity of cryopreserved human primary hepatocytes cultivated in a perfused multiwell device. Xenobiotica 45, 29–44. 10.3109/00498254.2014.944612 25068923

[B187] Vorrink S. U. Ullah S. Schmidt S. Nandania J. Velagapudi V. Beck O. (2017). Endogenous and xenobiotic metabolic stability of primary human hepatocytes in long-term 3D spheroid cultures revealed by a combination of targeted and untargeted metabolomics. FASEB Journal 31, 2696–2708. 10.1096/fj.201601375R 28264975 PMC5434660

[B188] Vorrink S. U. Zhou Y. Ingelman-Sundberg M. Lauschke V. M. (2018). Prediction of drug-induced hepatotoxicity using long-term stable primary hepatic 3D spheroid cultures in chemically defined conditions. Toxicol. Sci. 163, 655–665. 10.1093/toxsci/kfy058 29590495 PMC5974779

[B189] Walker T. M. Rhodes P. C. Westmoreland C. (2000). The differential cytotoxicity of methotrexate in rat hepatocyte monolayer and spheroid cultures. Toxicol. in Vitro 14, 475–485. 10.1016/S0887-2333(00)00036-9 10963964

[B190] Wang W. W. Khetani S. R. Krzyzewski S. Duignan D. B. Obach R. S. (2010). Assessment of a micropatterned hepatocyte coculture system to generate major human excretory and circulating drug metabolites. Drug Metab. Disposition 38, 1900–1905. 10.1124/dmd.110.034876 20595376

[B191] Wang J. D. Douville N. J. Takayama S. ElSayed M. (2012). Quantitative analysis of molecular absorption into PDMS microfluidic channels. Ann. Biomed. Eng. 40, 1862–1873. 10.1007/s10439-012-0562-z 22484830

[B192] Wang Z. Luo X. Anene-Nzelu C. Yu Y. Hong X. Singh N. H. (2015). HepaRG culture in tethered spheroids as an *in vitro* three-dimensional model for drug safety screening. J. Appl. Toxicol. 35, 909–917. 10.1002/jat.3090 25512232

[B193] Wang J. Chen F. Liu L. Qi C. Wang B. Yan X. (2016). Engineering EMT using 3D micro-scaffold to promote hepatic functions for drug hepatotoxicity evaluation. Biomaterials 91, 11–22. 10.1016/j.biomaterials.2016.03.001 26994875

[B194] Wang Y. I. Carmona C. Hickman J. J. Shuler M. L. (2018). Multiorgan microphysiological systems for drug development: strategies, advances, and challenges. Adv. Healthcare Mater. 7, 1–29. 10.1002/adhm.201701000 PMC5805562 29205920

[B195] Ware B. R. Khetani S. R. (2017). Engineered liver platforms for different phases of drug development. Trends Biotechnol. 35, 172–183. 10.1016/j.tibtech.2016.08.001 27592803 PMC5253249

[B196] Ware B. R. Berger D. R. Khetani S. R. (2015). Prediction of drug-induced liver injury in micropatterned co-cultures containing iPSC-derived human hepatocytes. Toxicol. Sci. 145, 252–262. 10.1093/toxsci/kfv048 25716675

[B197] Ware B. R. McVay M. Sunada W. Y. Khetani S. R. (2017). Exploring chronic drug effects on microengineered human liver cultures using global gene expression profiling. Toxicol. Sci. 157, 387–398. 10.1093/toxsci/kfx059 28369597

[B198] Wheeler S. E. Clark A. M. Taylor D. P. Young C. L. Pillai V. C. Stolz D. B. (2014). Spontaneous dormancy of metastatic breast cancer cells in an all human liver microphysiologic system. Br. J. Cancer 111, 2342–2350. 10.1038/bjc.2014.533 25314052 PMC4264444

[B199] Wilkening S. Stahl F. Bader A. (2003). Comparison of primary human hepatocytes and hepatoma cell line Hepg2 with regard to their biotransformation properties. Drug Metab. Disposition 31, 1035–1042. 10.1124/dmd.31.8.1035 12867492

[B200] Williams D. P. Shipley R. Ellis M. J. Webb S. Ward J. Gardner I. (2013). Novel *in vitro* and mathematical models for the prediction of chemical toxicity. Toxicol. Res. 2, 40–59. 10.1039/C2TX20031G PMC4765367 26966512

[B201] Wu Y. Lin Z. Y. W. Wenger A. C. Tam K. C. Tang X. S. (2018). 3D bioprinting of liver-mimetic construct with alginate/cellulose nanocrystal hybrid bioink. Bioprinting 9, 1–6. 10.1016/j.bprint.2017.12.001

[B202] Xu J. J. Henstock P. V. Dunn M. C. Smith A. R. Chabot J. R. de Graaf D. (2008). Cellular imaging predictions of clinical drug-induced liver injury. Toxicol. Sci. 105, 97–105. 10.1093/toxsci/kfn109 18524759

[B203] Yang Y. Wang K. Gu X. Leong K. W. (2017). Biophysical regulation of cell behavior-cross talk between substrate stiffness and nanotopography. Engineering 3, 36–54. 10.1016/J.ENG.2017.01.014 29071164 PMC5653318

[B204] Yin J. Meng Q. Zhang G. Sun Y. (2009). Differential methotrexate hepatotoxicity on rat hepatocytes in 2-D monolayer culture and 3-D gel entrapment culture. Chemico-Biological Interactions. 180, 368–375. 10.1016/j.cbi.2009.04.004 19394316

[B205] Yokoyama Y. Sasaki Y. Terasaki N. Kawataki T. Takekawa K. Iwase Y. (2018). Comparison of drug metabolism and its related hepatotoxic effects in HepaRG, cryopreserved human hepatocytes, and HepG2 cell cultures. Biol. Pharmaceut. Bull. 41, 722–732. 10.1248/bpb.b17-00913 29445054

[B206] Yu H. Barrass N. Gales S. Lenz E. Parry T. Powell H. (2015). Metabolism by conjugation appears to confer resistance to paracetamol (acetaminophen) hepatotoxicity in the cynomolgus monkey. Xenobiotica 45, 270–277. 10.3109/00498254.2014.973000 25335570

[B207] Zeilinger K. Schreiter T. Darnell M. Söderdahl T. Lübberstedt M. Dillner B. (2011). Scaling down of a clinical three-dimensional perfusion multicompartment hollow fiber liver bioreactor developed for extracorporeal liver support to an analytical scale device useful for hepatic pharmacological *in vitro* studies. Tiss. Eng. Part C: Methods 17, 549–556. 10.1089/ten.tec.2010.0580 21210724

